# Spatiotemporally Controlled Tumor Photodynamic/Immunotherapy Therapy Based on Upconversion Hybrid Nanosystem

**DOI:** 10.1002/advs.202515052

**Published:** 2025-11-21

**Authors:** Fang Wang, Wenfei Xu, Yuechen Liu, Shuxuan Zhu, Wenjing Liu, Shuhui Bo, Hong Sun, Bei Liu, Zhaogang Sun, Hongqian Chu

**Affiliations:** ^1^ Translational Medicine Center Beijing Chest Hospital Capital Medical University & Beijing Tuberculosis and Thoracic Tumor Research Institute Beijing 101149 China; ^2^ College of Science Minzu University of China Beijing 100081 China

**Keywords:** immunotherapy, photodynamic therapy, tumor, upconversion nanoparticles

## Abstract

Immunotherapy holds great promise for cancer treatment, but its clinical application is hindered by the immunosuppressive tumor microenvironment (TME) and the systematic toxicity. Herein, a spatiotemporally controllable smart nanodevice based on upconversion nanoparticles (UCNPs) that can be activated by dual near ‐ infrared (NIR) light for tumor immunotherapy, namely PURH, is developed. PURH is constructed by a five‐layer UCNPs surface coated with mesoporous silica, which encapsulates the photosensitizer rose bengal (RB) within the mesoporous channels. Additionally, CpG oligonucleotides (CpG ODN) is linked to the surface, followed by the surface functionalization of hyaluronic acid (HA) to achieve tumor targeting. PURH allows for dual NIR light activation, where 980 nm NIR light irradiation activates photodynamic therapy (PDT), inducing apoptosis and immunogenic cell death (ICD), while 808 nm NIR light irradiation triggers the release of CpG for tumor immunotherapy. The study offers a powerful tool and strategy for spatiotemporal controlled tumor immunotherapy.

## Graphical abstract

PURH is constructed by a five‐layer upconversion nanoparticle (UCNP) surface coated with mesoporous silica, which encapsulates rose bengal (RB) within the mesoporous channels. Additionally, CpG oligonucleotides (CpG ODN) is linked to the surface, followed by the surface functionalization of hyaluronic acid (HA). This spatiotemporally controllable smart nanodevice exhibits good performance of targeted tumor immunotherapy.

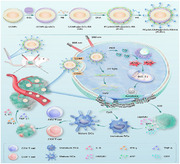

## 1 Introduction

Photodynamic therapy (PDT) has attracted more significant attention as an effective cancer treatment, due to its advantages, including high selectivity, non‐invasive, and spatio‐temporal control.^[^ ^1^, ^2^, ^3^ ^]^ PDT combines photosensitizers (PSs) and oxygen to generate reactive oxygen species (ROS), which ablate tumor cells under laser irradiation.^[^ ^4^ ^]^ Importantly, PDT can also induce immunogenic cell death (ICD), thereby enhancing the efficacy of anti‐tumor immunotherapy.^[^ ^5^ ^]^ However, the hypoxic tumor microenvironment (TME) exacerbated by PDT, along with limited light penetration depth, significantly hinders its therapeutic effectiveness against solid tumors.^[^ ^6^, ^7^ ^]^ Additionally, the immunosuppressive nature of TME complicates the reliance on PDT and its ICD for complete tumor ablation in many cases.^[^ ^8^, ^9^, ^10^ ^]^ Therefore, the combination of immunotherapy to enhance the antitumor immune response of PDT is a promising therapeutic approach.

In recent years, tumor immunotherapy has emerged as an efficient and promising tumor treatment strategy, capable of eradicating tumor cells or transforming the immunosuppressive TME by activating the host's own immune system.^[^ ^11^, ^12^ ^]^ Studies have shown that combining immune adjuvants with PDT can enhance the adaptive immune response following PDT, ultimately aiming for complete tumor cell eradication.^[^ ^13^ ^]^ Non‐methylated CpG oligodeoxynucleotide, a toll‐like receptor 9 (TLR‐9) agonist, is known to trigger both innate and adaptive immune responses and induce cytokine secretion by priming immature dendritic cells (iDCs).^[^ ^14^, ^15^ ^]^ However, free CpG requires multiple injections or high doses to effectively stimulate DCs maturation, and its efficacy is often limited by low cellular uptake, enzyme degradation, and potential toxicity from cytokine storms.^[^ ^16^, ^17^ ^]^ While various strategies for delivering CpG,^[^ ^18^, ^19^ ^]^ the challenge remains that the immunostimulatory activity of immune adjuvants cannot be precisely controlled. Achieving exogenous temporal and spatial control of immune responses is a critical issue in antitumor immunotherapy.^[^ ^20^ ^]^ Upconversion nanoparticles (UCNPs) can absorb two or more photons and emit visible and UV light under near‐infrared (NIR) excitation.^[^ ^21^, ^22^ ^]^ Due to their unique optical properties, UCNPs can activate photo‐responsive systems can be activated under NIR light.^[^ ^23^, ^24^, ^25^ ^]^ Therefore, it is essential to design a nanoplatform based on UCNPs that allows for specific control and regulation of immunotherapy with high spatiotemporal and precision, synergizing with PDT to achieve comprehensive therapeutic effects.

**Scheme 1:**
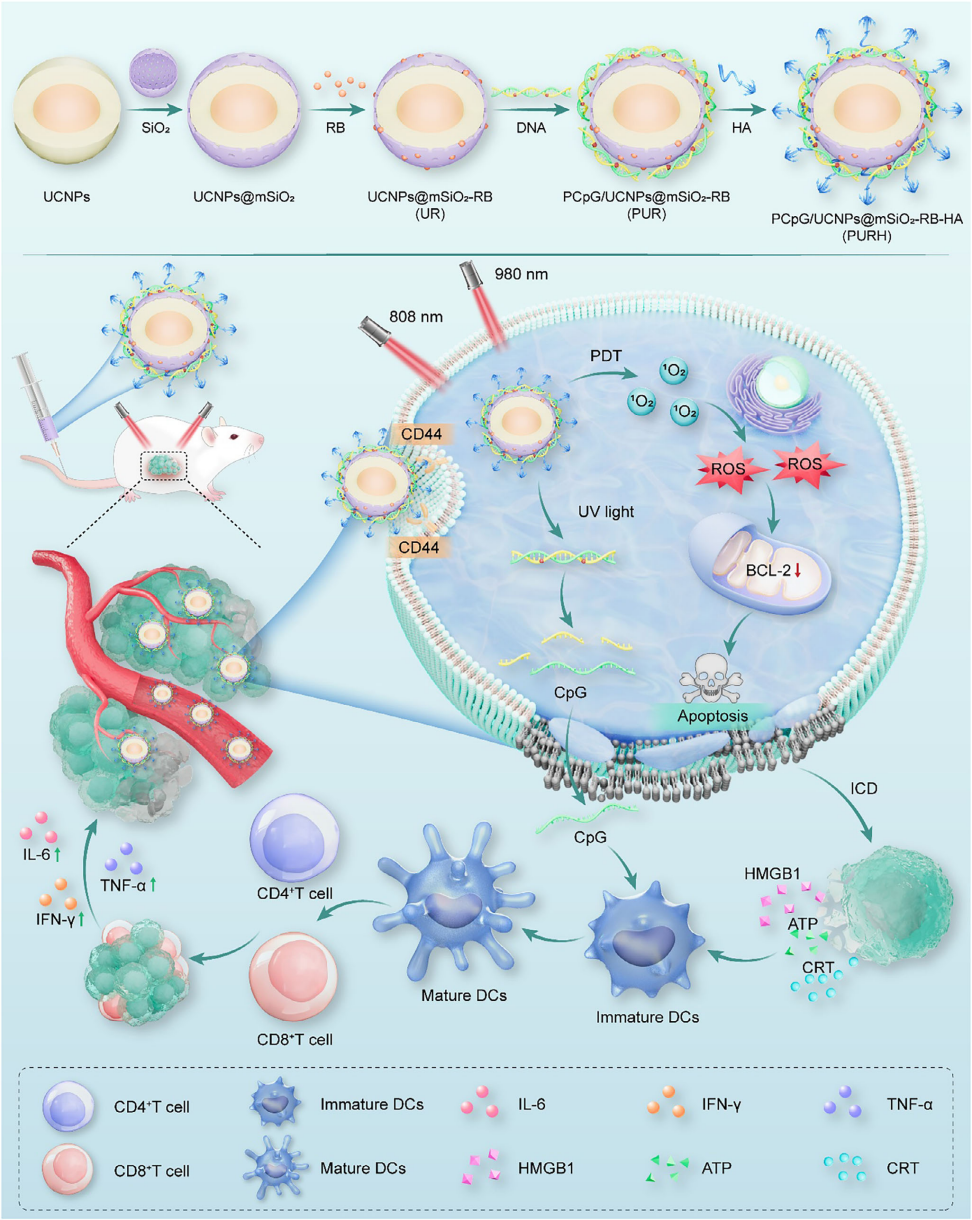
Schematic illustration of the preparation process and the mechanism of immunotherapy and PDT synergy therapy of the PCpG/UCNP@mSiO_2_‐RB‐HA (PURH) nanoplatform.

Herein, we designed a NIR light‐activated immune nanodevice based on an up‐conversion nanosystem that integrates PDT and an immune adjuvant into a tumor vaccine. This approach aims to enhance the therapeutic effect of PDT by boosting the host antitumor immune response with high spatiotemporal controllability (**Scheme** **1**). Mesoporous silicon modified UCNPs were loaded with photosensitizer rose bengal (RB) and designed to include reasonably designed UV‐activated CpG (PCpG) along with hyaluronic acid (HA)coating (PCpG/UCNP@mSiO_2_‐RB‐HA, PURH). Upon exposure to 980 nm light irradiation, the PURH system generates ROS and induces ICD to destroy the primary tumor. Due to the unique properties of UCNPs, they can convert 980 nm NIR light into UV–vis light, enabling the precise release of the immune adjuvant CpG through the cleavage of the photocleavable (PC) bonds in the complementary chain. This capability effectively reduces systemic toxicity while stimulating DCs maturation and T cell infiltration, ultimately enhancing anti‐tumor immunotherapy. Overall, the novel nanosystem facilitates temporal and spatial control of immunotherapy combined with PDT, thereby improving the systemic anti‐tumor therapy effects induced by PDT and highlighting its strong potential for clinical application.

## 2 Results and Discussion

### 2.1 Preparation and Characterization of PURH

**Figure 1:**
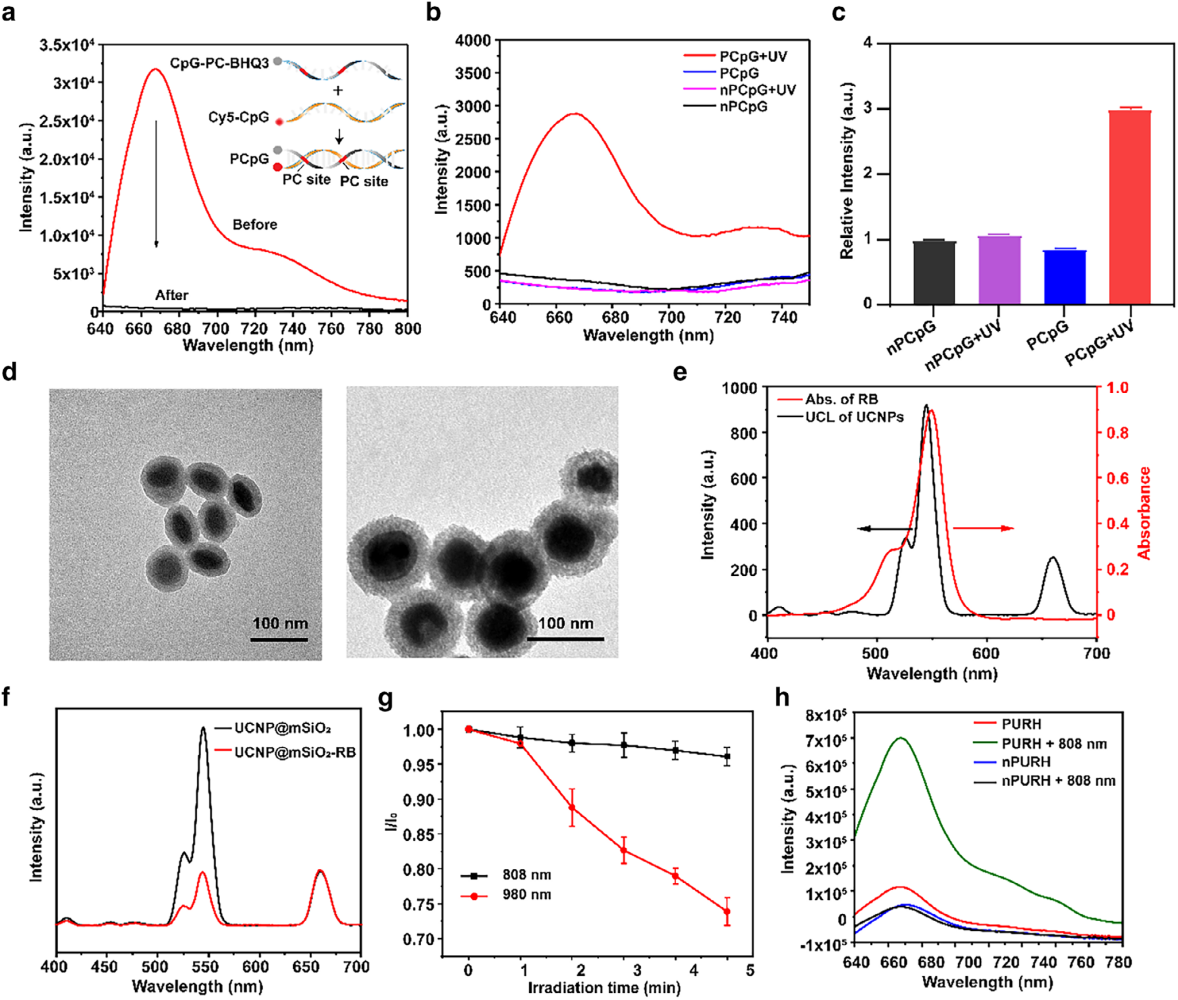
Characterizations of PURH nanosystem. a) Fluorescence spectra of Cy5 before and after the formation of PCpG. Inset: illustration of PCpG composite diagram. b,c) Fluorescence spectra (b) and the fluorescence intensity (c) of PCpG and nPCpG with and without UV irradiation. Data are presented as mean ± SD (n = 3). d) TEM images of UCNPs@mSiO_2_ and PURH. Scale, 100 nm. e) UCL spectrum of the UCNPs under 980 nm excitation and the UV–vis absorption spectrum of RB. f) UCL spectra of UCNPs@mSiO_2_ before and after loading with RB under 980 nm NIR light excitation. g) Normalized absorbance of DPBF in the presence of PURH under 980 nm or 808 nm NIR light irradiation. h) Fluorescence spectra of Cy5 in PURH solution after 808 nm NIR light irradiation for 10 min.

PCpG was synthesized through the hybridization of Cy5‐labeled CpG with BHQ3‐labeled cCpG modified by two PC bonds. The photoactivation properties of PCpG were evaluated using Förster resonance energy transfer (FRET).^[^ ^25^ ^]^ The fluorescence intensity of Cy5 was significantly decreased, confirming the successful synthesis of PCpG (**Figure** **1**a). Upon irradiation with UV light (UV, 365 nm, 5 mW cm^−2^), the PC bonds of PCpG were cleaved, leading to the release of Cy5‐labeled CpG and a subsequent increase in fluorescence intensity (Figure 1b,c; Figure S1, Supporting Information). To further validate the photo‐control mechanism of PCpG, we synthesized a contrasting structure, nPCpG, which has the same sequence as PCpG but lacks the PC bond. We observed no significant change in fluorescence intensity after UV irradiation. Additionally, the fluorescence intensity of Cy5 in double‐stranded PCpG progressively increased with the extension of UV light irradiation time (Figure S2, Supporting Information).

We synthesized oleic acid‐coated core‐multishell‐structure UCNPs (NaGdF_4_:Yb, Er@NaYF_4_@NaYF_4_:Yb, Tm@NaYbF_4_:Nd@NaYF_4_).^[^ ^26^ ^]^ Transmission electron microscopy (TEM) revealed that the synthesized UCNPs exhibited a uniform hexagonal plate‐like shape (Figure S3a–f, Supporting Information). Subsequently, mesoporous silica was coated onto the surface of the UCNPs to obtain UCNPs@mSiO_2_ (Figure 1d). High­angle annular dark­field scanning TEM (HAADF‐STEM), along with mapping and energy dispersive spectroscopy (EDS) line scan further confirmed the successful coating of mesoporous silica, which facilitates the loading of the photosensitizer RB into the hole (Figures S4 and S5, Supporting Information). The Er^3+^/Yb^3+^ co‐doped in the core of UCNPs generates green emission at 522 and 541 nm, as well as red emission at 656 nm under 980 nm NIR light irradiation. Meanwhile, Tm^3+^/Yb^3+^/Nd^3+^ co‐doped in the second and third shells produce UV emission at 347 and 363 nm and visible blue emission at 452 and 475 nm under 808 nm NIR light irradiation (Figure S6, Supporting Information). Next, UCNPs were modified with cationic polymers to facilitate the loading of PCpG onto their surface. We calculated the loading efficiencies of CpG and pCpG on UCNP@mSiO_2_‐RB to be 78.9% and 70.5%, respectively. Finally, an appropriate amount ofHA was added to form the HA layer, resulting in the synthesis of PCpG/UCNPs@mSiO_2_‐RB‐HA (PURH) with good dispersion (Figure 1d).

The green Upconversion luminescence  (UCL) band of UCNPs overlaps well with the absorption spectrum of RB under 980 nm NIR light irradiation (Figure 1e). Consequently, after loading RB into the mesoporous, the green UCL of UCNPs was significantly quenched (Figure 1f). The Zeta potential of PURH decreased to −18.4 mV following DNA functionalization and HA loading (Figure S7a, Supporting Information). The successful synthesis of PURH was further confirmed through dynamic light scattering (DLS) and the identification of characteristic absorption peaks of the different materials (Figure S7b,c, Supporting Information). 1, 3‐diphenylisobenofuran (DPBF), an indicator of single oxygen (^1^O_2_), was utilized to assess the ability of PURH to produce ^1^O_2_ under 980 or 808 nm NIR light irradiation. The results showed that the absorption intensity of DPBF decreased significantly under 980 nm NIR light irradiation, while no significant change was observed under 808 nm NIR light irradiation (Figure 1g). To investigate the photo‐controlled activation of PURH in solution, we observed that the fluorescence intensity of PURH increased under 808 nm NIR light irradiation, whereas no significant fluorescence change was noted in nPURH (Figure 1h). In addition, PURH demonstrated good stability in HEPES, PBS, and RPMI 1640 medium (Figure S8, Supporting Information).

### 2.2 In Vitro Photo‐Controlled Activation and Cytotoxicity of PURH

**Figure 2:**
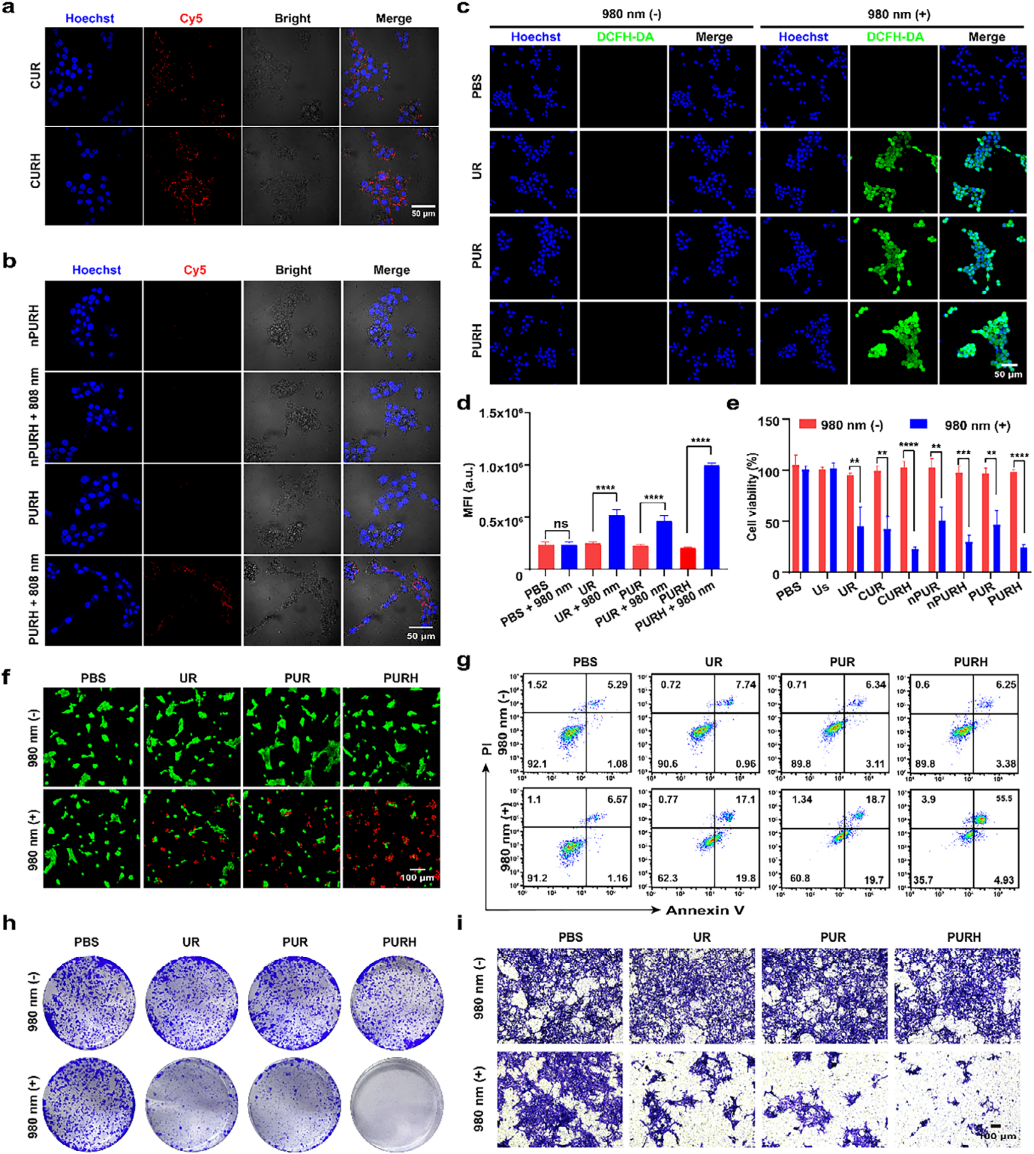
Intracellular uptake, photo‐controlled activation, and cytotoxicity of PURH in 4T1 cells. a) CLSM images of 4T1 cells treated with CUR and CURH (CpG labeled with Cy5). Scale bar, 50 µm. b) CLSM images of MCF‐7 cells treated with FRET pair‐labeled nPURH and PURH with or without 808 nm NIR light irradiation. Scale bar, 50 µm. c) The ROS production ability of different treatments with without 980 nm NIR light irradiation in 4T1 cells. Scale bar, 50 µm. d) The quantities analysis of ROS production in 4T1 cells by flow cytometry. Data are expressed as means ± SD (n = 3). e) Cell viability of 4T1 cells incubated with different treatments with or without 980 nm NIR light irradiation. Data are expressed as means ± SD (n = 4). f) CLSM images of Calcein‐AM (green) and PI (red) assays of 4T1 cells treated with different samples with and without 980 nm NIR light irradiation. Scale bar, 50 µm. g) Annexin V/PI double staining of cell apoptosis by flow cytometry. h) Colony formation assay of 4T1 cells with different treatments. i) Transwell migration assay of 4T1 cells with different treatments. **p < 0.01, ***p < 0.001, ****p < 0.0001, ns, no significance.

CpG is a well‐known potential immune adjuvant^[^ ^27^ ^]^ that can stimulate TLR‐9 to induce an immune response.^[^ ^28^ ^]^ We found that the cellular uptake capacity of CpG/UCNPs@mSiO_2_‐RB (CUR) was significantly stronger than that of free CpG (CpG strand was labeled with Cy5) in both 4T1 and MCF‐7 cells (Figure S9, Supporting Information). This enhanced uptake may be attributed to issues such as rapid nuclease degradation and low cellular uptake of free CpG.^[^ ^29^ ^]^ Furthermore, the cellular uptake capacity of PCpG/UCNPs@mSiO_2_‐RB (PUR) was also significantly greater than that of free PCpG (Figure S10, Supporting Information). HA can enhance the targeting of nanoparticles to various tumor cells, such as 4T1 and MCF‐7 cells, via CD44 receptors.^[^ ^30^ ^]^ To assess the cell targeting ability of CURH (CpG strand was labeled with Cy5), we employed a confocal laser scanning microscope (CLSM) and flow cytometry. As shown in **Figures** **2**a and S11 (Supporting Information), the Cy5 fluorescence intensity of CURH in 4T1 cells was significantly stronger than that of CUR, and this result was also confirmed in MCF‐7 cells (Figure S12, Supporting Information). We further investigated the photoactivated ability of PCpG in vitro. The PURH‐treated group (where Cy5‐labeled CpG hybridized with BHQ3‐labeled cCpG to form PCpG) exhibited significant fluorescence signals when irradiated with 808 nm NIR light, while no fluorescence changes were observed in the nPURH group (Figure 2b; Figure S13, Supporting Information). Additionally, there were no changes in fluorescence signals for either PURH or nPURH treated groups without 808 nm NIR light irradiation. These results indicated that the presence of 808 nm NIR light irradiation and PC bonds play crucial roles in the photo‐controlled activation of PCpG.

The ROS production ability of PURH in different breast cancer cells was further evaluated using 2, 7‐dichlorodihydrofluorescein diacetate (H_2_DCFDA). The results indicated that the PURH‐treated group produced more ROS than the PUR‐treated group with 980 nm NIR light irradiation in both 4T1 and MCF‐7 cells (Figure 2c; Figure S14, Supporting Information). Flow cytometry analysis also confirmed that the ^1^O_2_ level in the PURH‐treated group was 2.1‐fold higher than that in the PUR‐treated group with 980 nm NIR light irradiation (Figure 2d; Figure S15, Supporting Information). The results from the cell counting kit­8 (CCK‐8) assay showed that the cell viability in the PURH‐treated group (24.5%) was significantly lower than that of PUR‐treated group (46.6%) under 980 nm NIR light irradiation, and it was comparable to that of CURH treatment group (22.9%) (Figure 2e). Additionally, the cytotoxicity of PURH was not significantly different from that of nPURH. These results suggested that nanomaterials loaded with single‐ or double‐stranded CpG can induce similar cytotoxicity in 4T1 cells when irradiated with 980 nm NIR light. Similarly, MCF‐7 cells treated with PURH exhibited comparable cytotoxicity to that observed in 4T1 cells (Figure S16a, Supporting Information). Importantly, normal cells treated with different concentrations of PURH showed no significant cytotoxicity (Figure S16b, Supporting Information), further confirming the good biosafety of PURH.

The cytotoxicity of PURH was further evaluated by AM/PI assay and annexin V/PI analysis. The results demonstrated that PURH treatment following 980 nm NIR light irradiation can induce significant cell death (Figure 2f; Figure S17, Supporting Information). Flow cytometry analysis showed that the apoptosis rate induced by PURH was significantly higher than that of the PUR group under 980 nm NIR light irradiation (Figure 2g; Figure S18, Supporting Information). The expression of B‐cell leukemia/lymphoma 2 (BCL‐2), which is closely related to apoptosis,^[^ ^31^ ^]^ was assessed through western blot analysis. The results showed that the BCL‐2 expression level in the PURH‐treated group with 980 nm NIR light irradiation was significantly lower than that in the PUR and UR treatment groups (Figure S19, Supporting Information). Additionally, colony formation assay and transwell migration assays were conducted to further evaluate the anti‐proliferation and anti‐migration capabilities of PURH. As shown in Figure 2h,i, the number of cell clones in the PURH‐treated group was significantly reduced following 980 nm NIR light irradiation, and the cell migration ability was also markedly decreased. These results further confirm that PURH exhibits a good photodynamic effect and significantly promotes apoptosis in tumor cells.

### 2.3 In Vitro Activation of Antitumor Immune Responses of PURH

**Figure 3:**
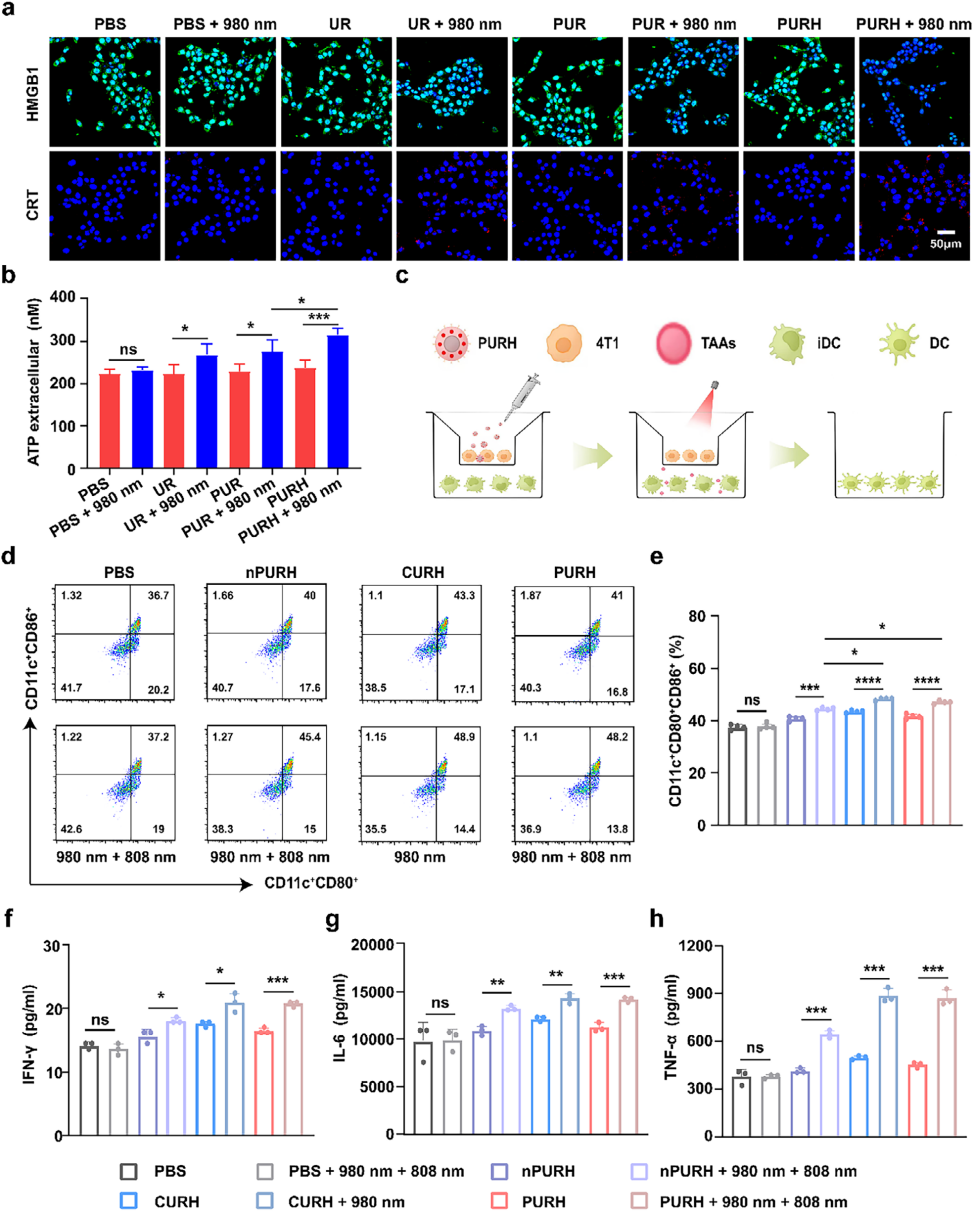
In vitro antitumor immune response induced by PURH. a) Immunofluorescence images of HMGB1 and CRT expression in 4T1 cells. Scale, 50 µm. b) The level of ATP release by 4T1 cells. Data are expressed as means ± SD (n = 3). c) Schematic illustration of the coculture system. d,e) Representative flow cytometry plots of matured DCs (CD11c^+^CD80^+^CD86^+^) after different treatments and the quantitative analysis of DCs maturation. Data are expressed as means ± SD (n = 4). f–h) Secretion levels of IFN‐γ, IL‐6, and TNF‐α in matured DCs suspensions. Data are expressed as means ± SD (n = 3). *p < 0.05, **p < 0.01, ***p < 0.001, ****p < 0.0001, ns, no significance.

ICD is a specific form of cell death that releases damage‐associated molecular patterns (DAMPs) to trigger an immune response.^[^ ^32^, ^33^, ^34^ ^]^ Calreticulin (CRT) translocation, high mobility group protein (HMGB1) and adenosine triphosphate (ATP) release were typical indicators of ICD.^[^ ^35^, ^36^ ^]^ Numerous studies have reported that PDT can induce ICD.^[^ ^37^, ^38^, ^39^ ^]^ To investigate the ability of PURH to induce ICD in vitro, we examined the expression of CRT and HMGB1, as well as the release of ATP following different treatments, with or without light irradiation. Compared with other groups, both 4T1 and MCF‐7 in the PURH‐treated group with 980 nm NIR light irradiation exhibited elevated CRT levels, while HMGB1 expression in the nucleus was significantly reduced (**Figure** **3**a; Figure S20, Supporting Information). The expression of HMGB1 was further evaluated using western blot analysis (Figure S21, Supporting Information), confirming the immunofluorescence staining results. Additionally, we assessed the secretion level of extracellular ATP. The results indicated that the ATP content in the PURH + 980 nm group was significantly higher than that in the PUR + 980 nm group and the PURH group (Figure 3b). Overall, the PDT of PURH effectively stimulates the release of DAMPs, thereby inducing ICD in vitro.

Tumor cells undergoing ICD can produce abundant tumor‐associated antigens (TAAs), which subsequently induced the maturation of iDCs within the TME.^[^ ^40^, ^41^, ^42^, ^43^, ^44^ ^]^ Therefore, we investigated the ability of PURH to induce the maturation of iDCs in vitro using a transwell system. As shown in Figure 3c, 4T1 cells were incubated in the upper chamber after different treatments, and then iDCs were incubated together in the lower chamber for 24 h. Following staining with DCs maturation markers (CD11c, CD80, and CD86), the maturation of DCs was assessed by flow cytometry. The percentage of DCs maturity (CD11c^+^CD80^+^CD86^+^) in the PURH + 980 nm + 808 nm group (48.2%) was higher than that in the nPURH + 980 nm + 808 nm group (45.4%), (Figure 3d,e). Notably, the percentage of DCs maturation in the CURH + 980 nm group (48.9%) was higher than that in the CURH group (43.3%). In contrast, the percentage of DCs maturation in the nPURH or PURH groups without 980 and 808 nm NIR light irradiation was lower. These results indicated that PURH can activate DCs maturation under combined 980 and 808 nm NIR light irradiation.

Mature DCs can secrete various immune‐related cytokines to regulate other immune‐ cells.^[^ ^45^, ^46^, ^47^ ^]^ Enzyme‐linked immunosorbent assay (ELISA) was employed to analyze the levels of immune‐related proinflammatory cytokines (TNF‐α, IL‐6, and IFN‐γ) secreted by DCs in the medium. As shown in Figure 3f–h, the expression levels of these cytokines in the PURH + 980 + 808 nm group were higher than those in the other groups. These results suggested that the release of DAMPs was induced by PDT, along with the photo‐controlled release of the immune adjuvant CpG of PURH, promotes DCs and subsequent cytokine production.

### 2.4 In Vivo Biological Distribution and Photo‐Controlled Activation

**Figure 4:**
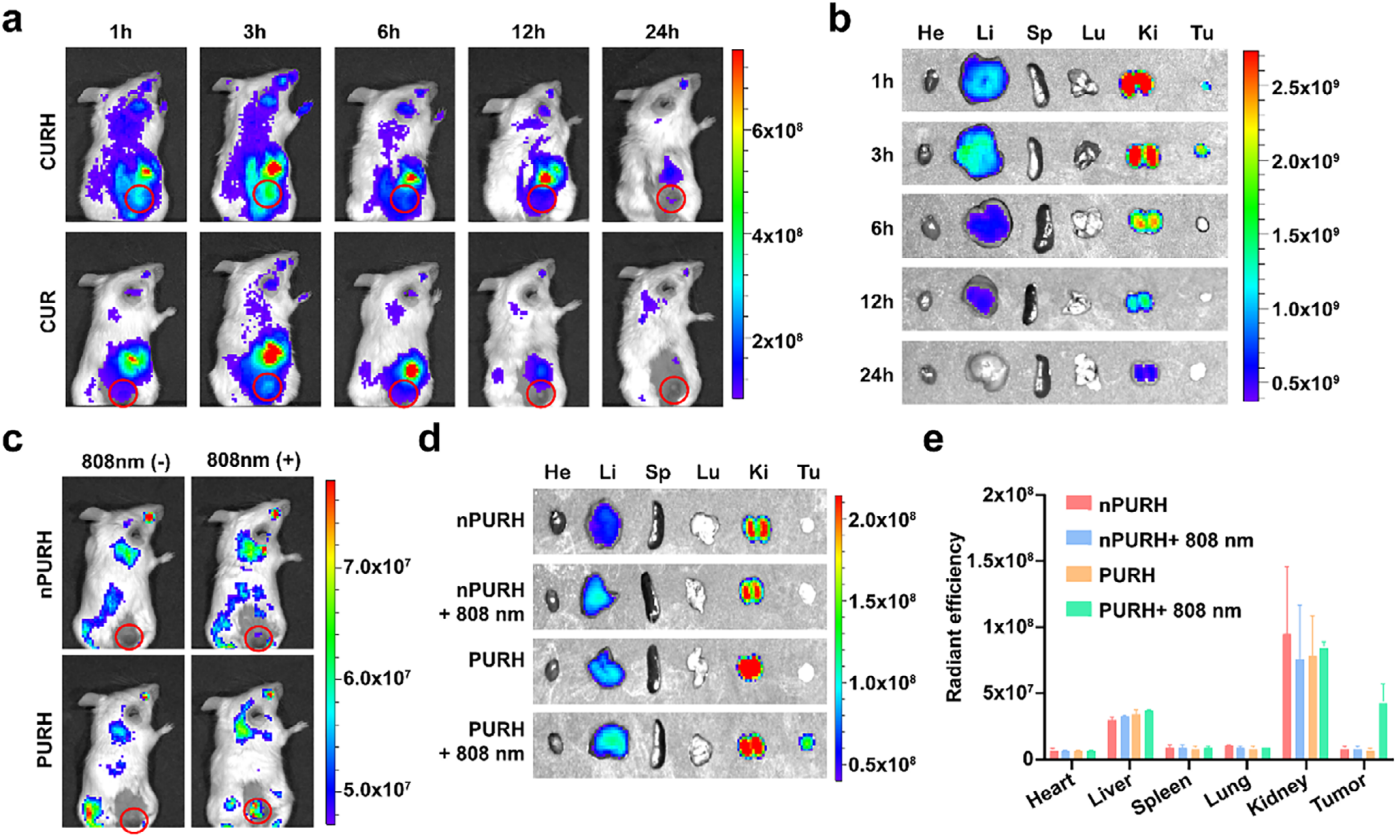
In vivo fluorescence imaging and photo‐controlled activation of PURH nanosystem. a) Representative whole‐body fluorescence images of 4T1 tumor‐bearing mice injected intravenously with CUR and CURH at the tumor site. Tumors are indicated by red circles. b) The quantitative analysis of fluorescence intensities at tumor sites in the panel. Data are presented as the mean ± SD (n = 4). c) Real‐time fluorescence imaging of representative tumor‐bearing mice after i.v. injection of FRET pair‐labeled nPURH or PURH followed with or without 808 nm irradiation. Tumors were indicated with red circles (left). d) *Ex vivo* fluorescence images of major organs and tumors from mice after different treatments. From left to right: heart, liver, spleen, lung, kidney, and tumor. e) The quantitative analysis of Cy5 fluorescence intensity in different organs. Data are presented as mean ± SD (n = 3).

To investigate the biological distribution of CURH, we administered CUR and CURH (Cy5‐labeled CpG) to mice by tail vein injection. The organs and tumors of mice were imaged at different time points (1, 3, 6, 12, and 24 h) post‐injection. As shown in **Figures** **4**a,b and S22a (Supporting Information), the fluorescence signal at the tumor site in the CURH‐treated group reached a peak at 3 h after injection. Furthermore, the fluorescence signal of the CURH‐treated group in tumor tissues was significantly stronger than that of the CUR group (Figure S22b, Supporting Information), indicating that hyaluronic acid‐modified CURH could significantly enhance tumor accumulation capacity.

To verify the photo‐controlled activation of PURH in vivo, FRET‐labeled PURH and nPURH were intravenously injected into tumor‐bearing mice with or without 808 nm NIR irradiation at the tumor site. The intratumoral fluorescence signal in the PURH + 808 nm group was significantly increased, as the 808 nm NIR light irradiation disconnected the PC site, releasing Cy5‐labeled CpG and restoring the fluorescence signal. In addition, the imaging results of tumors and normal organs in vitro were consistent with those observed in vivo (Figure 4c–e).

### 2.5 In Vivo Antitumor Efficacy

**Figure 5:**
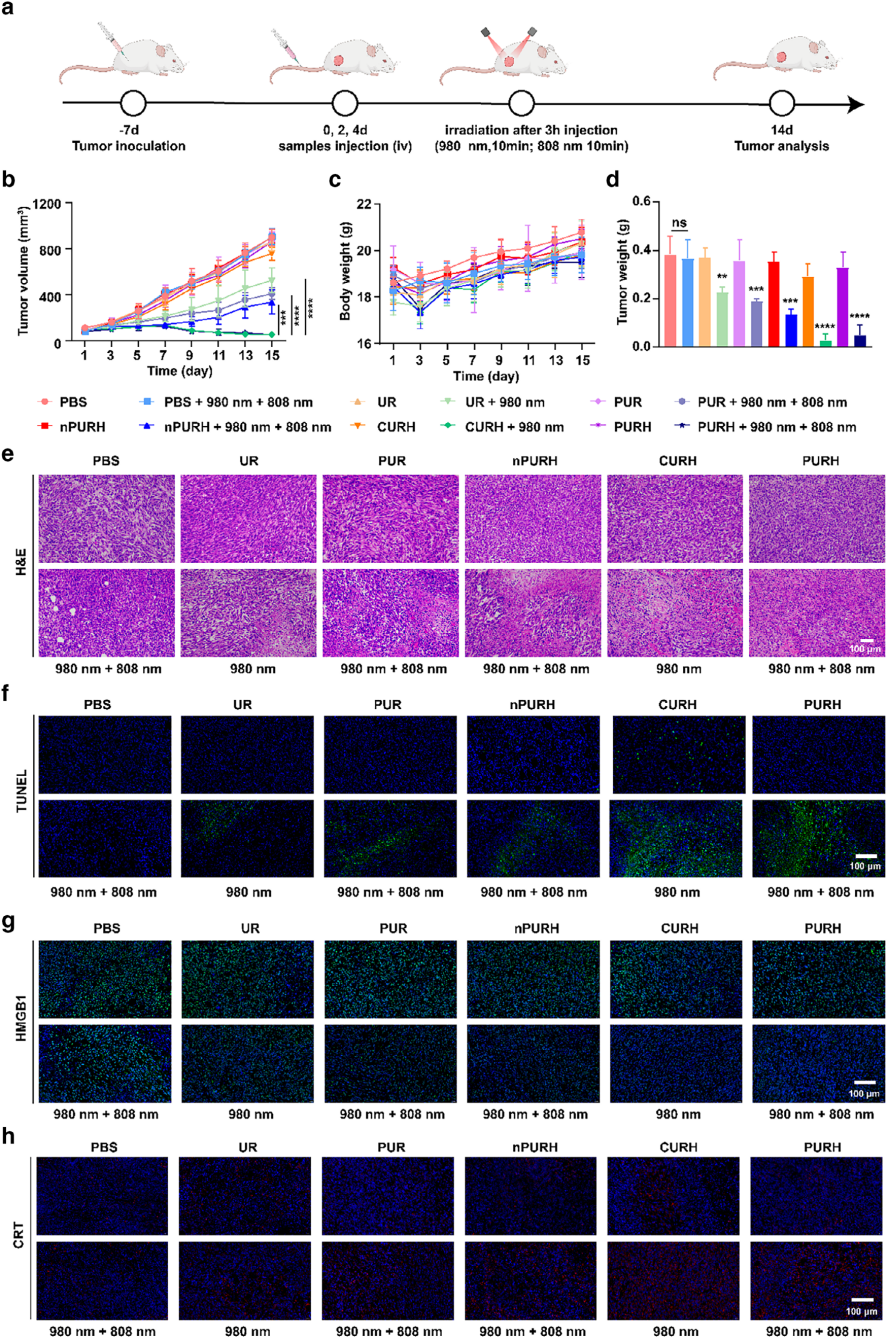
The effect of antitumor treatment of PURH in vivo. a) Schematic illustration of the antitumor experimental design in vivo. b) Tumor volumes of the different treatment groups at different times. Data are expressed as mean ± SD (n = 5). c) Tumor weights in the different treatment groups. Data are presented as mean ± SD (n = 5). d) Body weight of 4T1 tumor‐bearing mice under different treatments. Data are expressed as mean ± SD. (n = 5). e,f) H&E and TUNEL staining of tumor sections after different treatments. Scale bars, 100 µm. g,h) Immunofluorescence images of tumor sections stained by CRT and HMGB1. Scale bars, 100 µm. **p < 0.01, ***p < 0.001, ****p < 0.0001, ns, no significance.

We constructed 4T1‐tumor‐bearing BALB/C mice to evaluate the antitumor effect of PURH in vivo (**Figure** **5**a). The tumor‐bearing mice were randomly divided into 12 groups and given corresponding treatment: PBS, PBS + 980 nm + 808 nm, UR, UR + 980 nm, PUR, PUR + 980 nm + 808 nm, nPURH, nPURH + 980 nm + 808 nm, CURH, CURH + 980 nm, PURH, PURH + 980 nm + 808 nm. NIR activation was performed 3 h postinjection by irradiating the tumor regions with 980 and 808 nm NIR lights for 10 min (1.2 W cm^−2^, 1 min irradiation, and 5 min break). Mice weight and tumor volume were monitored every other day, and the mice were euthanized after 2 weeks. As shown in Figure 5b,c and Figure S23 (Supporting Information), the PURH + 980 nm + 808 nm group and the CURH + 980 nm group significantly inhibited tumor growth. In addition, the tumor inhibition effect of the PURH + 980 nm + 808 nm group was significantly stronger than that of the PUR + 980 nm + 808 nm and nPURH + 980 nm + 808 nm groups. This enhanced effect was attributed to the synergistic action of the PURH group, leveraging HA targeting and CpG‐induced immune response to boost antitumor efficacy. These results indicate that photo‐controlled PURH can produce a synergistic therapeutic effect through PDT and immunotherapy. There were no significant differences in tumor growth among the UR, PUR group, nPURH group, or PURH groups compared to the PBS group. However, the tumor volume and weight in the CURH group were significantly lower than those in the PBS group, which may be due to the immune response elicited by CpG ODN. During the treatment period, the body weight of mice across different treatment groups was negligible (Figure 5d). Furthermore, hematoxylin and eosin (H&E)staining and terminal deoxynucleotidyl transferase‐mediated deoxyuridine triphosphate nick end labeling (TUNEL)staining results showed that the PURH + 980 nm + 808 nm group induced more tumor tissue necrosis and apoptosis (Figure 5e,f). PURH can induce ICD and release DAMPs with light irradiation in vitro, thereby enhancing the anti‐tumor effect. Therefore, we further analyzed the expression of HMGB1 and CRT in vivo using immunofluorescence to evaluate the ability of PURH to induce ICD. As shown in Figure 5g,h, the PURH + 980 nm + 808 nm group significantly increased CRT expression in the cytoplasm, while decreased HMGB1 expression in nucleus within tumor tissues. These findings further supported the application of PURH under NIR light irradiation to induce ICD in tumor cells via PDT and immunotherapy.

To further evaluate the biosafety of PURH, major organs (heart, liver, spleen, lung, and kidney) of mice were collected forH&E staining after treatment. The H&E staining of liver from the CURH‐treated group showed significant damage and inflammation compared to the PURH‐treated group (Figure S24, Supporting Information). There were no significant tissue damage or pathological changes in the heart, lung, spleen, and kidney of mice in different treatment groups (Figure S25, Supporting Information). In addition, the biochemical indices alanine aminotransferase (ALT) and aspartate aminotransferase (AST) in the CURH group were higher than those in the other groups (Figure S26, Supporting Information). Complete blood counts remained normal in all different treatment groups (Figure S27, Supporting Information). These results indicated that PURH not only significantly inhibited tumor growth, but also did not induce liver toxicity, demonstrating that PURH has good biological safety. In addition, we performed a blood hemolysis experiment to confirm the hemolytic effect of PURH on blood cells (Figure S28, Supporting Information). The results showed that PURH was generally safe for blood cells.

### 2.6 In Vivo Activation of Antitumor Immunity

**Figure 6:**
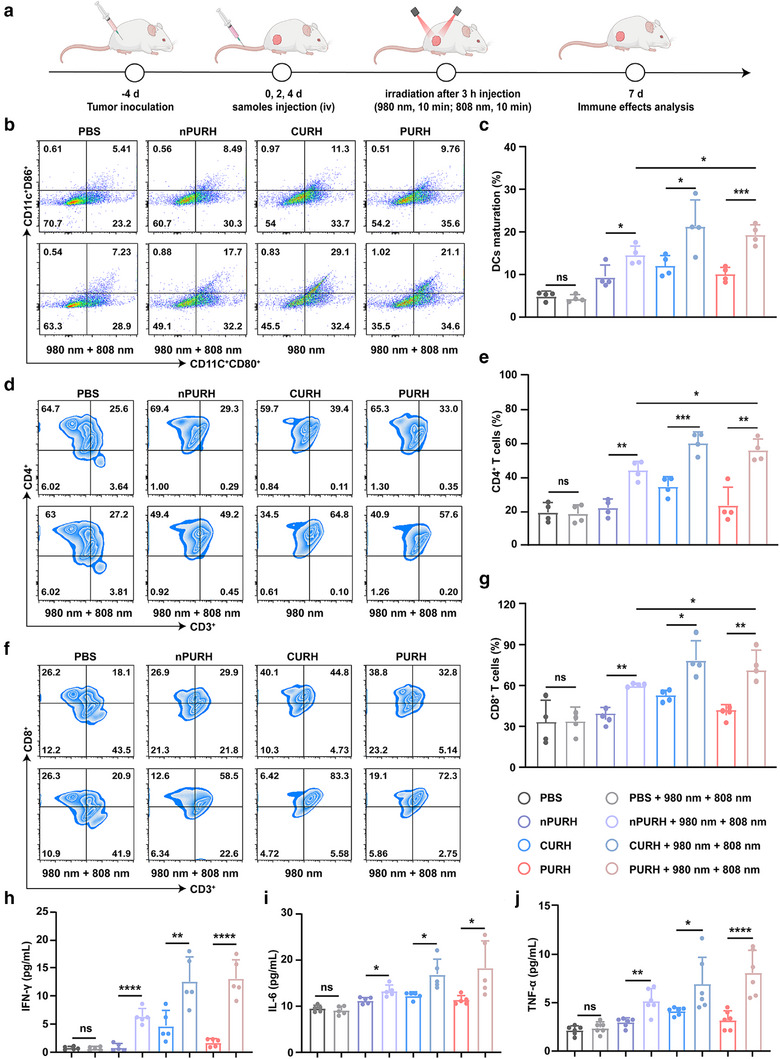
In vivo antitumor immune responses induced by PURH. a) Schematic illustration of the antitumor experimental design in vivo. b) Representative flow cytometry plots of matured DCs (CD11c^+^CD80^+^CD86^+^) in tumor following different treatments. c) Percentages of matured DCs in tumors. Data are presented as means ± SD (n = 4). d) Representative flow cytometry plots of CD4^+^ T cells (CD3^+^CD4^+^) in tumors following different treatments. e) Percentages of CD4^+^ T cells in tumors. Data are presented as means ± SD (n = 4). f) Representative flow cytometry plots of CD8^+^ T cells (CD3^+^CD8^+^) in tumors following different treatments. g) Percentages of CD8^+^ T cells in tumors. Data are presented as means ± SD. (n = 4). h–j) The levels of IFN‐γ, IL‐6, and TNF‐α in the serum of 4T1 tumor‐bearing mice after different treatments. Data are presented as means ± SD (n = 4). *p < 0.05, **p < 0.01, ***p < 0.001, ****p < 0.0001, ns, no significance.

DAMPs induced by ICD can promote the maturation of DCs and enhance the infiltration of helper T lymphocytes (CD4^+^ T cells) and cytotoxic T lymphocytes (CD8^+^ T cells) into solid tumors, transforming the immunosuppressive TME into an immunoreactive microenvironment.^[^ ^48^, ^49^ ^]^ To further evaluate the immune response in vivo, BALB/c tumor bearing mouse model was treated with PBS, PBS + 980 nm+ 808 nm, nPURH, nPURH + 980 nm + 808 nm, CURH, CURH + 980 nm, PURH, PURH + 980 nm + 808 nm, respectively (**Figure** **6**a). NIR activation was performed 3 h postinjection by irradiating the tumor regions with 980 and 808 nm NIR lights for 10 min (1.2 W cm^−2^, 1 min irradiation, and 5 min break). On day 7 after intravenous injection, the subcutaneous tumors were resected for single‐cell suspension preparation. The maturation of DCs (CD11c^+^CD80^+^CD86^+^) was analyzed using flow cytometry. As shown in Figure 6b,c, the DCs maturation rate of the nPURH + 980 nm + 808 nm group (17.7%) was significantly higher than that of the PBS group (5.41%). In addition, the proportion of CD11c^+^CD80^+^CD86^+^ cells in the PURH + 980 nm + 808 nm group was significantly higher than that in the nPURH + 980 nm + 808 nm group, indicating that PURH can effectively induce DCs maturation through the synergistic effect of PDT and immunotherapy.

We also examined the infiltration of CD4^+^ T cells and CD8^+^ T cells to further verify the systemic immune response. Flow cytometry analysis showed that the infiltration of CD4^+^ T cell and CD8^+^ T cell, respectively, increased to 49.2% and 58.5%, respectively, in the nPURH + 980 nm + 808 nm group compared with the PBS group (Figure 6d–g). Furthermore, in the PURH + 980 nm + 808 nm group, the infiltration of CD4^+^ T cells and CD8^+^ T cells increased to 57.6% and 72.3%, respectively, which was not statistically different from that observed in the CURH + 980 nm group. We also assessed the proportion of regulatory T cells (Tregs) in the immune microenvironment and found that Treg infiltration decreased by 43.1% after PURH + 980 nm + 808 nm treatment compared with PBS (Figure S29, Supporting Information). These results indicated that the synergistic effect of PDT and immunotherapy of PURH significantly enhances the infiltration of CD4^+^ T cells and CD8^+^ T cells and reduced the infiltration of Tregs, promoting a robust systemic anti‐tumor immune response and effectively eradicate tumors.

To further understand the synergistic effect of PURH based on PDT and immunotherapy, we analyzed the changes in cytokines (IFN‐γ, IL‐6, and TNF‐α) in the serum of mice after treatment using ELISA. We observed that the serum from the PURH + 980 nm + 808 nm group showed significantly enhanced secretion of inflammatory cytokines compared to the other groups, indicating that PURH induced a strong immune response (Figure 6h–j; Figure S30, Supporting Information). These results confirm that the synergistic strategy of PDT and immunotherapy employed by PURH demonstrates excellent potential for anti‐tumor immunotherapy.

## 3 Conclusion

In recent years, PDT has demonstrated significant potential as an effective tumor treatment, capable of inhibiting tumor growth by destroying localized tumors and activating systemic antitumor immune responses. Importantly, PDT can induce ICD in tumor cells, further contributing to tumor suppression. However, most ICD induced by PDT often fails to produce sufficient antitumor immune responses to completely eradicate tumor cells.^[^ ^50^, ^51^ ^]^ Therefore, a synergistic treatment strategy combining PDT and immunotherapy provides an innovative approach to clinical cancer treatment. In this work, we propose a DNA‐based NIR light triggering strategy that induces ROS production under NIR light while allowing for the temporally and spatially controlled release of CpG. The synergistic mechanism of PDT and immunotherapy in PURH can be summarized as follows: 1) Under 980 nm NIR light irradiation, PURH activates photosensitizer RB to produce ROS, thereby inducing ICD and enhancing the antitumor immune response. 2) PURH effectively delivers redesigned CpG ODN, protecting them from degradation and improving cellular uptake. 3) The unique optical properties of UCNPs enable the disconnection of the PC site of CpG complementary chain under 808 nm NIR light irradiation, facilitating a high spatial and temporal release of CpG that minimizes systemic toxicity. Moreover, the released CpG stimulates DCs maturation and promotes T cell infiltration, synergizing with PDT to enhance the antitumor effect. These findings suggest that the PURH nanoplatform serves as a non‐invasive nanodevice that can provide a safe and effective treatment strategy for clinical antitumor immunotherapy.

## 4 Experimental Section

### Materials and Reagents

Rare earth oxides, oleic acid (OA, 90%), 1‐octadecene (ODE, 95%), and oleylamine (OM, 90%) were obtained from Acros. Trifluoroacetic acid (99%), APTES, TEOS, and Sulfo‐SMCC were purchased from Sigma–Aldrich. Dulbecco's modified Eagle's medium (DMEM), RPMI 1640 medium, and fetal bovine serum (FBS) were obtained from Wisent Bio Co., Ltd. (Nanjing, China). Phosphate buffered saline (PBS), trypsin, and penicillin–streptomycin were purchased from EallBio Biomedical Technology Co., Ltd. (Beijing, China). Opti‐MEM was purchased from Gibco (California, USA). DCFH‐DA, Hoechst 33342, and ROS Assay Kit obtained from Solarbio (Beijing, China). Calcein/AM double staining kit was obtained from Beyotime (Beijing, China). Annexin V/PI apoptosis kit was purchased from Elabscience (Wuhan, China). Cell Counting Kit‐8 (CCK‐8) was bought from NCM Biotech (Suzhou, China). All HPLC‐purified DNA molecules were synthesized by Sangon Biotech Co., Ltd. (Shanghai, China) (Table S1, Supporting Information). Brilliant Violet 510 anti‐mouse CD3, Alexa Fluor 700 anti‐mouse CD4, PerCP/Cyanine5.5 anti‐mouse CD8a, APC/Fire 750 anti‐mouse CD45, Alexa Fluor 488 anti‐mouse CD86, PE anti‐mouse CD80, and APC anti‐mouse CD11c were purchased from Biolegend (USA). Matrigel was purchased from Corning (USA). 35‐mm glass‐bottom dishes (Cellvis) were purchased from Beijing Haimabor Biotechnology Co., Ltd. (Beijing, China). The water used throughout the experiments was Millipore water (18.2 MΩ). All the chemicals were used as received without further purification.

### Instruments

The transmission electron microscopic (TEM) images were captured on the JEM‐1200EX transmission electron microscope (JEOL, Japan). The scanning electron microscope (SEM) images were obtained from the SU8220 scan electron microscope (Hitachi Co. Ltd, Japan). Fluorescence spectra were captured on an Edinburgh FS5C fluorimeter (UK). UV–vis spectra were recorded by an Edinburgh DS5 spectrophotometer (UK). Cell viability data were obtained using a Thermo MULTISCAN GO reader (Thermo, USA). The CLSM images were obtained using an Olympus FluoView FV1000 confocal microscope (Olympus Corporation, Japan). The flow cytometry assays were carried out using Beckman CytoFLEX (Beckman Coulter, USA). T cells and DCs analysis in tumors was using Attune flow cytometry (ThermoFisher, USA). The in vivo fluorescence images were obtained on an IVIS Spectrum in vivo imaging system (PerkinElmer Inc., USA).

### Synthesis of NaGdF_4_:Yb,Er UCNPs

Metal trifluoroacetate precursors (0.02 mmol Er(CF_3_COO)_3_, 0.2 mmol Yb(CF_3_COO)_3_, 0.78 mmol Gd(CF_3_COO)_3,_ and 1 mmol CF_3_COONa)) were added to a 100 mL three‐neck flask containing OA (10 mmol), OM (10 mmol), and ODE (20 mmol). The mixture was heated to 120 °C under intense stirring under vacuum to remove water and oxygen, and then heated to 310 °C and kept under nitrogen protection for 50 min. After cooling to room temperature, α‐phase NaGdF_4_:Yb,Er‐UCNPs was precipitated by adding ethanol, centrifugally collected, and re‐dispersed in 10 mL cyclohexane. The prepared nanoparticles (5 mL) were then added to a 100 mL three‐necking flask containing OA (20 mmol) and ODE (20 mmol) and metal trifluoroacetate precursors (0.01 mmol Er(CF_3_COO)_3_, 0.1 mmol Yb(CF_3_COO)_3_, 0.39 mmol Gd(CF_3_COO)_3,_ and 0.5 mmol CF_3_COONa). The mixture was heated to 120 °C and kept under vacuum for 30 min, and then heated to 310 °C and kept in a nitrogen atmosphere for 50 min. The synthesized β‐phase NaGdF_4_:Yb,Er‐UCNPs was precipitated by adding ethanol, centrifugally collected, and re‐dispersed in 10 mL cyclohexane.

### Synthesis of Core‐Multi‐Shell Structured UCNPs

The core‐multishell UCNPs (NaGdF_4_:Yb,Er@NaYF_4_@NaYF_4_:Yb,Tm@NaYbF_4_:Nd@NaYF_4_) synthesized continuous cladding using β‐phase NaGdF_4_:Yb,Er as crystal seeds. The prepared colloidal solution of NaGdF_4_:Yb,Er (5 mL) was added to a 100 mL three‐necked flask and heated at 120 °C for 30 min. The reaction mixture was then heated to 310 °C and kept under nitrogen protection for 50 min. The prepared NaGdF_4_:Yb,Er@NaYF_4_ UCNPs was collected by centrifugation, washed and redispersed in 10 mL cyclohexane. The shell precursors (0.02 mmol Tm(CF_3_COO)_3_, 0.9 mmol Yb(CF_3_COO)_3_, 0.08 mmol Y(CF_3_COO_3_)_3_ and 1 mmol CF_3_COONa) were used to synthesize NaGdF_4_:Yb,Er@NaYF_4_@NaYF_4_:Yb,Tm. The shell precursors (1 mmol Nd(CF_3_COO)_3_, 1 mmol Yb(CF_3_COO)_3_ and 2 mmol CF_3_COONa) were used to synthesize NaGdF_4_:Yb,Er@NaYF_4_@NaYF_4_:Yb,Tm@NaYbF_4_:Nd. Finally, NaGdF_4_:Yb,Er@NaYF_4_@NaYF_4_:Yb,Tm@NaYbF_4_:Nd@NaYF_4_ UCNPs were prepared with shell precursors (0.5 mmol Y(CF_3_COO)_3_ and 0.5 mmol CF_3_COONa).

### Preparation of PCpG/UCNPs@mSiO_2_‐RB‐HA (PURH)

First, 0.1 g CTAB was mixed with 20 mL deionized water and 1 mL UCNPs and then stirred overnight. Next, 40 mL deionized water, 6 mL ethanol, and 100 µL 2 mol sodium hydroxide were added to the mixture and the temperature was raised to 60 °C. The mixture was added to the ethanol solution of TEOS (200 µL TEOS and 1 mL ethanol) and stirred for 1 h. Then the APTES solution (100 µL APTES and 1 mL alcohol) was added, and the mixture was heated and stirred for 30–60 min. The resulting precipitate (UCNPs@mSiO_2_) was collected by centrifugation and dispersed in 2 mL of deionized water. 1 mL RB (1 mg mL^−1^) was mixed with 1 mL UCNPs@mSiO_2_ and stirred in the dark for 24 h, collected by centrifugation and dispersed in 1 mL deionized water to obtain UCNPs@mSiO_2_‐RB (UR). To prepare PCpG/UCNPs@mSiO_2_‐RB (PUR), PCpG and UR were stirred in 20 mm HEPES buffer (150 mm NaCl, 5 mm MgCl_2_, pH 7.4) at a molar ratio of 25:1 for 1 h. The obtained PUR was centrifuged, washed to remove the unattached PCpG, and redispersed in deionized water. Finally, PURH was prepared by the reaction of HA and PUR.

### Characterizations of PURH

Dynamic light scattering of Malvern Zetasizer Nano ZS (Zetasizer Nano‐ZSP, Malvern Instruments, UK) was used to determine particle size and Zeta potential of different nanomaterials. The morphological distribution of the particles was characterized by transmission electron microscopy (Jeol, Japan). The loading of DNA and RB was assessed by UV–vis spectroscopy (Edinburgh Instruments, England).

### Photo‐Controlled Activation Detection

PCpG was generated by hybridization of cCpG labeled with a BHQ3 quencher and PC site with Cy5‐modified CpG. Briefly, cCpG labeled with a BHQ3 quencher and PC site and Cy5‐CpG (100 µm in Millipore water) were diluted to 20 µm in HEPES buffer. The mixture was heated (95 °C, 5 min) and then cooled to room temperature. PCpG (100 nm) was irradiated with UV (365 nm, 5 mW cm^−2^) for different times (0, 1, 2, 3, 4, 5, and 6 min) to detect the fluorescence intensity of Cy5.

### Gel Electrophoresis Analysis of Oligonucleotide Damage

The synthesized PCpG or nPCpG were irradiated with or without UV light (365 nm, 5 mW cm^−^^2^) for 5 min. The PAGE gel was prepared according to the polyacrylamide gel kit. The mixture of 6 × DNA loading and DNA double strands was added to the wells of the PAGE gel and electrophoresis was carried out at a voltage of 120 V for 1.5 h. The PAGE gel was stained with Gel Red dye and the images were collected by the gel imaging system.

### Singlet Oxygen Detection

The DMSO solution of DPBF (1 mg mL^−1^) was added to a cuvette containing PURH. Then, the corresponding absorption spectra were measured under dark conditions with 980 or 808 nm light irradiation (1.2 W cm^−2^).

### Stability Test of PURH

PURH in different buffers (20 mm HEPES, PBS, and RPMI 1640 medium + FBS) was centrifuged at 12 000 rpm for 10 min at different time points (0, 1, 2, 4, 6, and 8 h). The fluorescence changes were detected to determine the stability of PURH.

### Cellular Culture

MCF‐7 cells were cultured in DMEM with 10% FBS and 1% penicillin–streptomycin mixture, while 4T1 cells were cultured in RPMI 1640 with 10% FBS and 1% penicillin‐streptomycin mixture. Both cells were cultured in an incubator at 5% CO_2_ and 37 °C.

### Cellular Uptake

MCF‐7 or 4T1 cells were seeded into 35 mm glass‐bottom confocal dishes or six‐well plates and incubated for 12 h. Different nanomaterials modified with Cy5 (CUR, CURH, PUR, and PURH) were incubated for 4 h. After washing with PBS, the cells were fixed with 4% paraformaldehyde for 10 min. Finally, the cells were stained with Hoechst 33342 for 10 min, and the image was captured using CLSM. The cells inoculated in the six‐well plate were treated with different nanomaterials, and then the cells were collected and washed three times with PBS. The cells were re‐suspended with appropriate PBS and subsequently analyzed by flow cytometry.

### PURH Intracellular Light‐Controlled Activation

MCF‐7 or 4T1 cells were seeded in 35 mm glass‐bottom confocal culture dishes or six‐well plates and incubated overnight. After incubating with PURH and nPURH (CpG modified with Cy5) for 4 h, the cells were irradiated with or without 808 nm NIR light for 10 min (1.2 W cm^−2^, 1 min irradiation, and 5 min break). After washed with PBS, the cells were fixed with 4% paraformaldehyde, stained with Hoechst 33342, and then the fluorescence signal intensity of Cy5 in the cells was detected by CLSM.

### Cell Cytotoxicity Assay

MCF‐7 or 4T1 cells were seeded in 96‐well plates and incubated overnight. After switching to Opti‐MEM, the cells were treated with PBS, UR, CUR, CURH, nPUR, PUR, nPURH and PURH for 4 h. After irradiation with 980 nm NIR light for 10 min (1.2 W cm^−2^, 1 min irradiation, and 5 min break), the cells were replaced with normal medium for another 24 h. 10 µL CCK‐8 solution was added to each well and the incubation continued for 1 h. Finally, the absorbance value at 450 nm was measured using an enzyme‐labeled instrument. In addition, human normal lung epithelial cells Beas‐2b were incubated with different amounts of PURH for 24 h and CCK‐8 assays were performed according to the same procedure as above.

### ROS Detection

MCF‐7 or 4T1 cells were cultured on 35 mm glass‐bottom confocal culture dishes or 6‐well plates for 12 h. After the cells were treated with different nanomaterials (UR, PUR, and PURH) for 4 h, the cells were irradiated with 980 nm NIR light for 10 min (1.2 W cm^−2^, 1 min irradiation, and 5 min break). Then, the cells were washed twice in serum‐free medium and incubated with DCFH‐DA (10 µm) at 37 °C for 20 min. After washing with serum‐free medium and PBS, the nucleus was stained with Hoechst 33342 for 10 min, and then the images were collected by CLSM. In addition, 4T1 cells seeded in six‐well plates were treated according to the previous steps, digested, and centrifuged to collect the cells. After washing twice with PBS, the appropriate amount of PBS was added for re‐suspension, and the production of reactive oxygen species was detected by flow cytometry.

### Live/Dead Cell Analysis

MCF‐7 or 4T1 cells were seeded into 35 mm glass‐bottom confocal culture dishes and incubated for 12 h. After the cells were treated with PBS, UR, PUR, and PURH for 4 h, the cells were irradiated with 980 nm NIR light for 10 min (1.2 Wcm^−2^, 1 min irradiation, and 5 min break), and continued to incubate for 1 h. According to the instructions of the Calcein/PI cell activity and cytotoxicity detection kit, the cells were placed back in the incubator for 30 min under the condition of dark light, and finally the images were collected by CLSM.

### Cell Apoptosis

4T1 cells were seeded in 6‐well plates and incubated overnight. The cells were treated with Opti‐MEM medium containing PBS, UR, PUR, and PURH for 4 h, irradiated with 980 nm NIR light for 10 min (1.2 W cm^−2^, 1 min irradiation, and 5 min break), and continued to incubate for 1 h. After washing with PBS once, the cells were digested and centrifuged, then washed with PBS twice. The cell suspension was mixed with Annexin V‐FITC and PI and incubated for 15 min in the dark. Finally, the level of cell apoptosis was analyzed by flow cytometry.

### Western Blot

4T1 cells were seeded in 6‐well plates and incubated for 12 h. Then, the cells were treated with PBS, UR, PUR, and PURH for 4 h, and irradiated with 980 nm NIR light for 10 min (1.2 W cm^−2^, 1 min irradiation, and 5 min break). Replace with complete medium and continue incubation for 36 h. After washing with PBS, 200 µL of RIPA cracking buffer and PMSF mixture were added to each well, and lysed on ice for 20 min. The samples were centrifuged at 12 000 rpm for 15 min, and then the supernatants were absorbed, which were the total extracted protein. The BCA protein detection kit was used to determine the protein concentration. 40 µL of 5x loading buffer was added to each sample tube and thoroughly mixed. The samples were heated at 100 °C for 5 min and immediately placed on ice for cooling. The proteins were isolated using 10% SDS‐PAGE gel and subsequently transferred to nitrocellulose membrane. After blocking with milk powder for 1.5 h at room temperature, the nitrocellulose membrane was incubated overnight at 4 °C with primary antibodies against BCL‐2 (Immunoway, YM3041, 1:1000), HMGB1 (Immunoway, YM4697, 1:1000), and β‐actin (Immunoway, YM3028, 1:1000). On the second day, the NC membrane was incubated with HRP‐Goat anti‐mouse IgG (Immunoway, RS0001,1:5000) at room temperature for 1 h. Finally, the enhanced chemiluminescence solution was added for the reaction and the chemiluminescence instrument was used for detection.

### Immunofluorescence Staining

MCF‐7 or 4T1 cells were seeded in 35 mm glass‐bottom confocal culture dishes and incubated for 12 h. After the cells were treated with PBS, UR, PUR, and PURH for 4 h, the cells were irradiated with 980 nm NIR light for 10 min (1.2 W cm^−2^, 1 min irradiation, and 5 min break) and incubated overnight. The next day, the cells were fixed with 4% paraformaldehyde for 10 min, permeated with 0.1% Triton X‐100 for 10 min, and washed with PBS three times for 5 min each time. Then, the cells were blocked with 5% BSA for 1 h, stained with primary anti‐CRT (Abcam, ab196158, 1:500) and anti‐HMGB1 (1:200) at room temperature for 1 h or incubated at 4 °C overnight. The cells were re‐stained with Goat Anti‐Rabbit IgG H&L (Alexa Fluor 488) (Abcam, ab150077, 1:500) and incubated at room temperature for 1 h. Finally, the nuclei were stained with Hoechst 33342 for 10 min and the images were acquired by CLSM.

### Colony Formation Assay

4T1 cells were seeded in six‐well plates and incubated overnight. The cells were treated with PBS, UR, PUR, and PURH for 4 h, and then irradiated with 980 nm NIR light for 10 min (1.2 W cm^−2^, 1 min irradiation, and 5 min break). After being replaced with complete medium, the cells were cultured in the incubator for 12 h. The cells were digested and collected and then re‐suspended with RPMI 1640 medium and counted by using bovine Baud's counting plate. The cells were seeded in six‐well plates at a density of 800 cells/well and incubated in incubators. The medium was changed at intervals of 3–4 days. The cells were stopped incubation when the number of cells in the colony exceeded 50 or the cell colonies were visible to the naked eye. After the cells were washed twice with PBS, 4% paraformaldehyde was added to the plate and fixed for 20 min. After fixation, 1 mL crystal violet solution with a concentration of 0.1% was added and stained for 30 min. Finally, the six‐well plates were washed with tap water, dried, and kept in the dark. The images were collected, and the colony differences were counted and analyzed.

### Transwell Migration Assay

4T1 cells were seeded in six‐well plates and incubated overnight, and then the cells were treated with PBS, UR, PUR, and PURH for 4 h. After irradiated with 980 nm NIR light for 10 min (1.2 W cm^−2^, 1 min irradiation, and 5 min break), the cells were replaced with complete medium and cultured in the incubator for 12 h. The cells were digested and collected and then re‐suspended with RPMI 1640 medium and counted by using bovine Baud's counting plate. The cells were seeded in a six‐well plate at a density of 200 cells µL^−1^. 700 µL of medium containing 10% FBS was added to the lower chambers of the 24‐well plate, and then the upper chamber was slowly placed into the lower chamber. 200 µL cell suspension diluted with FBS‐free medium was absorbed and slowly added to the upper chambers. Subsequently, the 24‐well plate was incubated in an incubator for 36 h. After incubation, the upper chambers were cleaned with PBS and the walls of the upper chambers were gently wiped with cotton swabs. Add 1 mL of 4% paraformaldehyde to the lower chambers, put the upper chambers into it and fix for 30 min. Next, add 1 mL 0.1% crystal violet solution to the lower chambers, place the upper chambers in the lower chambers, and dye for 30 min. Subsequently, the upper chambers should be slowly rinsed with tap water. After natural drying, they should be properly stored in the dark. Finally, high‐power microscope was used to randomly collect images of 6–9 fields.

### Adenosine Triphosphate (ATP) Detection

4T1 cells were seeded in 12‐well plates and incubated overnight, and then treated with UR, PUR and PURH for 4 h. The cells were irradiated with 980 nm NIR light for 10 min and then replaced with complete medium for 24 h The cells were digested and collected, and 1 mL of the extract was added to reinsert them. Under ice bath conditions, the cell suspension was ultrasonically broken for 1 min (ultrasound for 2 s, and pause for 1 s), and then centrifuged (12 000 rpm) at 4 °C for 10 min. The supernatants were collected into another EP tube, and 500 µL chloroform was added to the tube. Then, they were thoroughly mixed and centrifuged at 12 000 rpm at 4 °C for 3 min. The supernatants were removed and placed on ice for testing. The standard solution of 10 µmol mL^−1^ ATP was diluted 16 times with distilled water to prepare a standard solution with a concentration of 0.625 µmol mL^−1^. Preparation of working liquid: reagent 2 (mL):reagent 3 (mL):reagent 4 (mL):reagent 5 (mL):reagent 6 (mL) = 1:1:0.1:1:0.4:0.1. In a 96‐well plate, the sample or standard liquid of 20 µL was added to the measuring tube and the standard tube, and then 128 µL reagent 1 and 52 µL working liquid were added. The absorption value A1 for 10 s was measured at 340 nm wavelength immediately after mixing. The 96‐well plate was placed in an incubator at 37 °C for 3 min. After the reaction, the absorption value A2 was rapidly determined at 3 min and 10 s. The difference between A1 and A2 of the sample tube and the standard tube was calculated, respectively, and the ATP content was obtained (ATP content (µmol L^−1^) = 0.125 × ΔA determination ÷ ΔA standard).

### Detection of Induced Maturation of Mice Bone Marrow‐Derived Dendritic Cells (BMDCs)

Immature BMDCs were obtained by inducing the prepared mouse bone marrow‐derived cells with Inaba modification method. The cell density was adjusted to 0.5–1×10^6^ mL^−1^ by adding an appropriate volume of RPMI 1640 complete medium containing 10% FBS, recombinant mouse GM‐CSF (20 ng mL^−1^) and IL‐4 (10 ng mL^−1^). 1 mL of cell suspension was added to each well of the 24‐well plate and continued to be cultured, which was labeled as day 0 of culture. The plate was gently shaken every two days and replaced with fresh medium containing cytokines by 3/4 of the original volume. On the 4th day of culture, BMDCs were observed to be attached to the bottom of the plate. On the 6th day, more BMDCs were observed to form colony growth. Suspended cells and loosely attached immature BMDCs were collected, and then appropriate amount of complete medium was added to adjust the cell density to 1 × 10^6^ mL^−1^. 1 mL of cell suspension was added to each well in a 24‐well plate and continued to culture for 24 h. 4T1 cells were digested and collected, and then re‐suspended with RPMI 1640 medium and counted, and then the cell density was adjusted to 250 cells µL^−1^. 700 µL of medium containing 10% FBS was added to the lower chambers of the 24‐well plate, and then the upper chamber was slowly placed into the lower chamber. 200 µL 4T1 cells suspension was absorbed and slowly added to the upper chambers. Subsequently, the 24‐well plate was incubated in an incubator overnight. 4T1 cells were treated with UR, PUR, and PURH for 4 h. The cells were irradiated with 980 nm NIR light for 10 min and then replaced with complete medium. The upper chambers cultured with 4T1 cells were transferred to the lower chambers containing BMDCs and incubated together for 24 h. BMDCs and the cell supernatants were collected, respectively.

Then, appropriate amounts of Cell Staining buffers were added to ensure that the cell density of cell suspension was 5–10 × 10^6^ mL^−1^, and 100 µL cell suspension was added to new flow tubes. 0.25 µg (100 µL) TruStain FcX PLUS was added to each tube and incubated for 10 min on ice in the dark. The flow surface staining antibodies (PE‐CD80, 0.5ug (100 µL); APC‐CD11c, 0.25µg (100 µL); FITC‐CD86, 1µg (100 µL)) were added to the cell suspension that had been blocked by FcR, mixed and incubated on ice for 3 min in the dark. After washing twice with Cell Staining Buffer, the cells were resuspended with 500 µL of Cell Staining Buffer, and then 5 µL of dead and alive dye 7‐AAD was added. After incubation for 10 min under dark conditions, the data were collected using flow cytometry. The cell supernatants described above were used to detect the expression of cytokines by ELISA kit.

### 4T1 Tumor‐Bearing Mice Model

The female BALB/c mice (16–18 g) were purchased from Beijing Vital River Laboratory Animal Technology Co., Ltd. The 4T1 tumor xenograft mice model was established by subcutaneous injection of 4T1 cells (1 × 10^6^ cells, 100 µL, 1:1 (V/V) PBS and Matrigel). The tumor volume (V) was calculated by using the formula V = ((Length × width^2^)/2), where “length” and “width” represent the longest and shortest lengths, respectively. All animal experiments were approved by the Experimental Animal Committee of Beijing Tuberculosis and Thoracic Tumor Research Institute.

### In Vivo Imaging and Light‐Controlled Activation Evaluation

When the tumor volume grew to about 120 mm^3^, the tumor‐bearing BALB/c mice were randomly divided into two groups (n = 5). CUR and CURH (Cy5‐labeled CpG, DNA dose of 25 nmol kg^−1^) were injected into tail vein, respectively. in vivo fluorescence imaging was performed on mice at different time points (1, 3, 6, 12, 24, and 48 h) after injection using the IVIS imaging system.

The 4T1 tumor‐bearing mice were randomly divided into two groups (n = 8), which were injected with FRET labeled nPURH and PURH (Cy5 labeled CpG, BHQ3 labeled the complementary chain of CpG) in the tail vein, respectively. Mice injected with the same drug were randomly divided into two groups (n = 4). Three hours after the injection, one group received 808 nm NIR light (1.2 W cm^−^^2^, 1 min irradiation, and 5 min break) to illuminate the tumor site for 10 min, while the other group received no light treatment. The mice were imaged in vivo using the IVIS imaging system, and tumors and major organs (heart, liver, spleen, lung, and kidney) were removed for in vitro fluorescence imaging analysis.

### Evaluation of Antitumor Efficacy In Vivo

When the tumor volume of BALB/c tumor‐bearing mice model reached ≈50 mm^3^, the mice were randomly divided into 12 groups (n = 5), and received different treatment treatments: PBS, PBS + 980 nm + 808 nm, UR, UR + 980 nm + 808 nm, PUR, PUR + 980 nm + 808 nm, nPURH, nPURH + 980 nm + 808 nm, CURH, CURH + 980 nm, PURH and PURH + 980 nm + 808 nm. Each mouse was injected three times (DNA dose was 25 nmol kg^−1^) through the tail vein every 2 days. Three hours after the injection, the tumor site was exposed to 980 nm or 980 nm + 808 nm NIR light (1.2 W cm^−2^, 1 min irradiation, and 5 min break) for 10 min. The tumor volume and body weight changes of mice in each group were measured and recorded regularly every two days. After 14 days, the mice were euthanized, and their tumor tissue was collected and weighed. The tumor tissue samples were fixed with 4% paraformaldehyde and subjected to paraffin embedding treatment. Subsequently, the sections were cut from the embedded tissues and treated with H&E staining and TUNEL staining, respectively.

### Evaluation of Antitumor Immunity In Vivo

When the tumor volume of BALB/c tumor‐bearing mice reached ≈80 mm^3^, the mice were randomly divided into 8 groups (n = 5): PBS, PBS + 980 nm + 808 nm, nPURH, nPURH + 980 nm + 808 nm, CURH, CURH + 980 nm, PURH and PURH + 980 nm + 808 nm. The mice were given corresponding treatments by tail vein injection (DNA dose of 25 nmol kg^−1^), with or without 980 nm or 980 nm + 808 nm NIR light irradiation (1.2 W cm^−2^, 1 min irradiation, and 5 min break). The injection was given once every other day for a total of three times. 48 h after the last injection, the mice were euthanized, and tumor tissue samples and serum samples were collected. The tumor tissues were washed clean and chopped, and then digested at 37 °C for 70 min with the digestive enzyme ‐Hanks solution (200 mL HBSS (containing Ca^2+^ and Mg^2+^) + 100 mg collagenase I + 20 mg hyaluronidase + 20 mg DNase). The tissue suspensions of different groups after digestion were ground and filtered into 50 mL centrifuge tubes (containing 70 µm filter screens), and then centrifuged at 2000 rpm for 5 min. After removing the supernatant, the red blood cells were lysed with ammonium chloride erythrocyte lysate. The samples were centrifuged and washed twice with PBS. The cells were resuspended by adding an appropriate amount of cell staining buffer and the cell density of each tube was adjusted to about 1×10^6^/100 µL. According to the fluorescent antibody staining with antibody instructions, respectively, the proportion of CD4^+^ T and CD8^+^ T cells and the maturation rate of dendritic cells were detected by flow cytometry. In addition, the expression of cytokines in serum samples was detected according to the instructions of the ELISA kit.

### Assessment of Biosafety and Biocompatibility

Healthy BALB/c mice were intravenously injected with PBS, UR, PUR, nPURH, CURH, and PURH, respectively (DNA dose of 25 nmol kg^−1^). After two weeks, the mice were euthanized, and the blood samples were collected. The collected whole blood samples were used for classification and counting of blood cells. The serum samples were subjected to biochemical analysis using an automatic biochemical analyzer. In addition, the main organs of mice (heart, liver, spleen, lung, and kidney) were collected and subjected to paraffin embedding treatment and H&E staining.

### Hemolysis Test of PURH

The blood compatibility of PURH at different concentrations was investigated by hemolysis assay. First, the blood from BALB/c mice was obtained and washed three times. The blood was co‐incubated with PURH (12.5, 25, 50, 100, and 200 µg mL^−1^) for 1 h. After the incubation, the solution was centrifuged, and the supernatant was separated to measure its absorption value by UV–vis. The PBS and water were used as negative and positive controls.

### Abbreviations

Abbreviations used in this paper are listed in Table S2 (Supporting Information).

### Statistical Analysis

All experiments were independently repeated at least three times, and the obtained data were all expressed as mean ± standard deviation. Statistical analysis was performed using Student's t‐test and one‐way analysis of variance. *p < 0.05, **p < 0.01, ***p < 0.001 and ****p < 0.0001 were considered statistically significant. All data were processed and analyzed using GraphPad prism 8 software.

## Conflict of Interest

The authors declare no conflict of interest.

## Supporting information



Supporting Information

## Data Availability

The data that support the findings of this study are available from the corresponding author upon reasonable request.
