# Supplementary material for: Spatiotemporally Controlled Tumor Photodynamic/Immunotherapy Therapy Based on Upconversion Hybrid Nanosystem
Source: Adv Sci (Weinh). 2025 Nov 21;13(7):e15052. doi: 10.1002/advs.202515052 (PMC12866702; doi:10.1002/advs.202515052)
Supplement: Supplementary file 1 — Supporting Information [file ADVS-13-e15052-s001.docx]

Supporting Information

**Spatiotemporally Controlled Tumor Photodynamic / Immunotherapy Therapy Based on Upconversion Hybrid Nanosystem**

*Fang Wang, Wenfei Xu, Yuechen Liu, Shuxuan Zhu, Wenjing Liu, Shuhui Bo, Hong Sun,* Bei Liu,* Zhaogang Sun,* Hongqian Chu,**


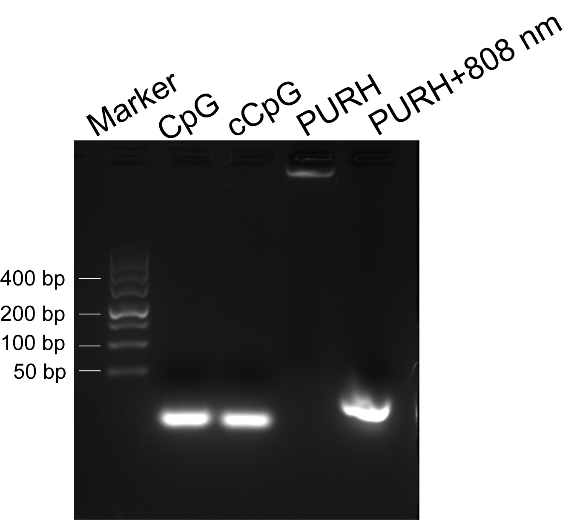


**Figure S1.** Agarose gel electrophoresis was used to examine the integrity of the PCpG construct and the release of CpG after 808 nm NIR light irradiation. Lane 1: free CpG; Lane 2: free cCpG; Lane 3: PURH; Lane 4: PURH +808 nm light irradiation.


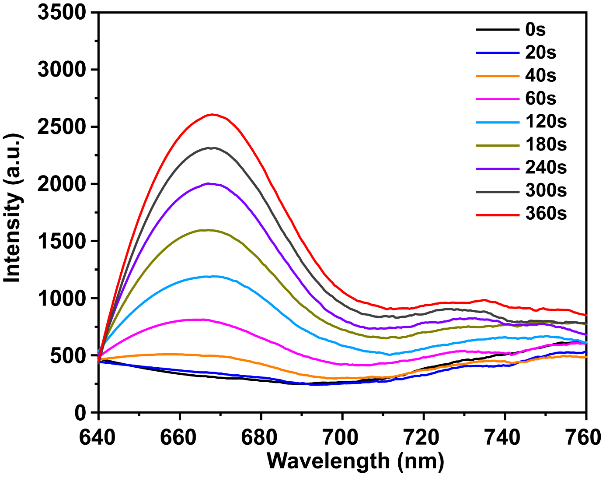


**Figure S2.** The fluorescence intensity changes of DNA double-stranded PCpG with the extension of ultraviolet light irradiation time.


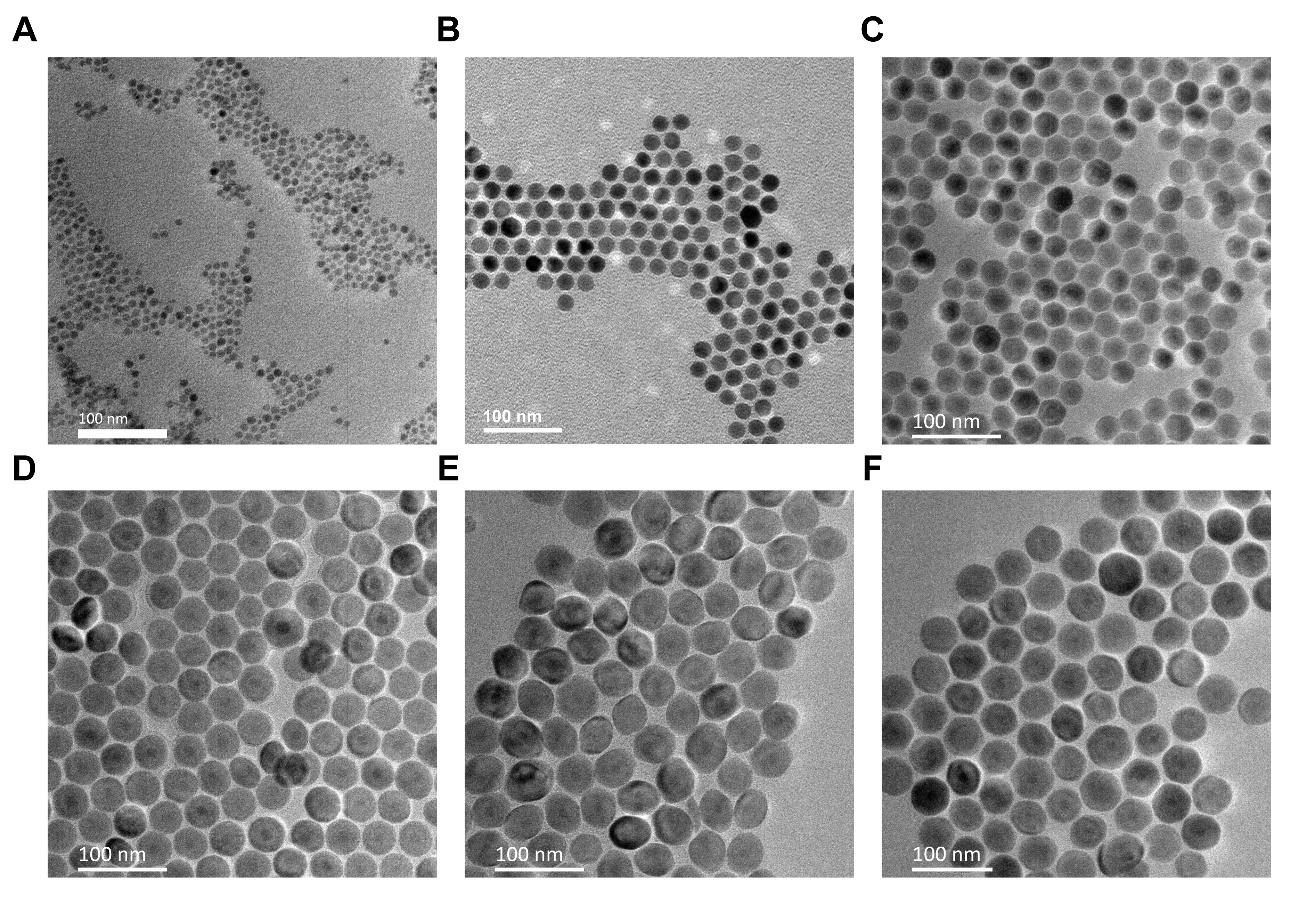


**Figure S3.** TEM image of α-phase NaGdF_4_:Yb,Er (a), β-phase NaGdF_4_:Yb,Er (b), NaGdF_4_:Yb,Er@NaYF_4_ (c), NaGdF_4_:Yb,Er@NaYF_4_@NaYF_4_:Yb,Tm (d) NaGdF_4_:Yb,Er@NaYF_4_@NaYF_4_:Yb,Tm@NaYbF_4_:Nd (e), and NaGdF_4_:Yb,Er@NaYF_4_@NaYF_4_:Yb,Tm@NaYbF_4_:Nd@NaYF_4_ UCNPs (f).


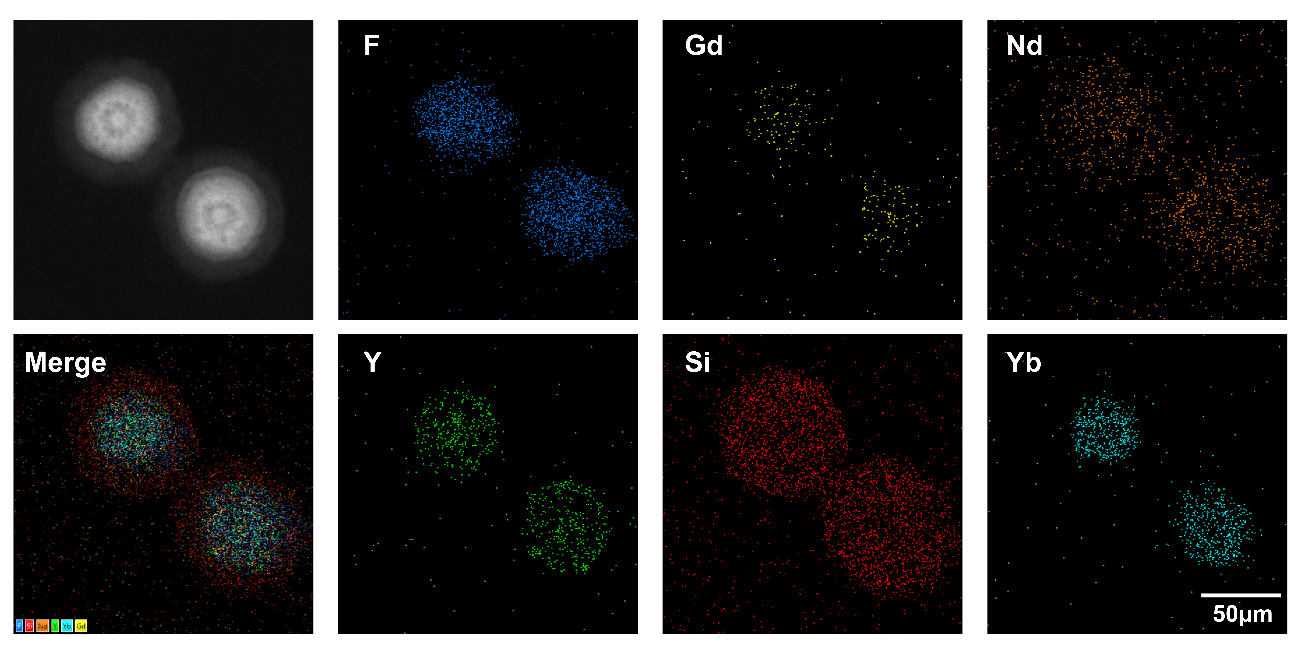


**Figure S4.** High-angle annular darkfield scanning transmission electron microscopy image and corresponding elemental mapping images of UCNPs@mSiO_2_.


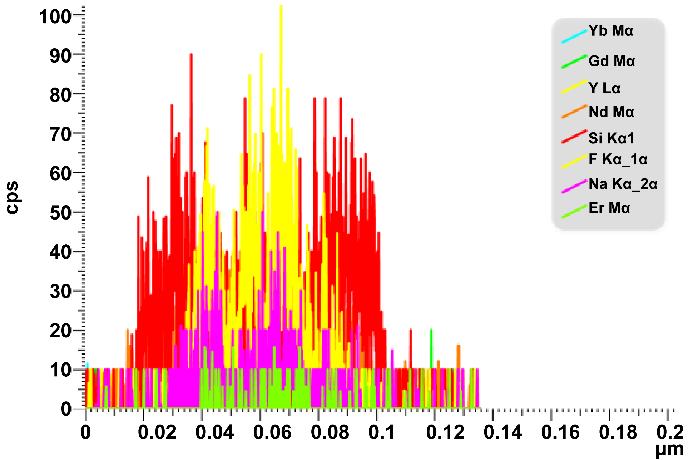


**Figure S5.** Energy dispersive spectroscopy (EDS) line scan profiles of UCNPs@mSiO_2_.


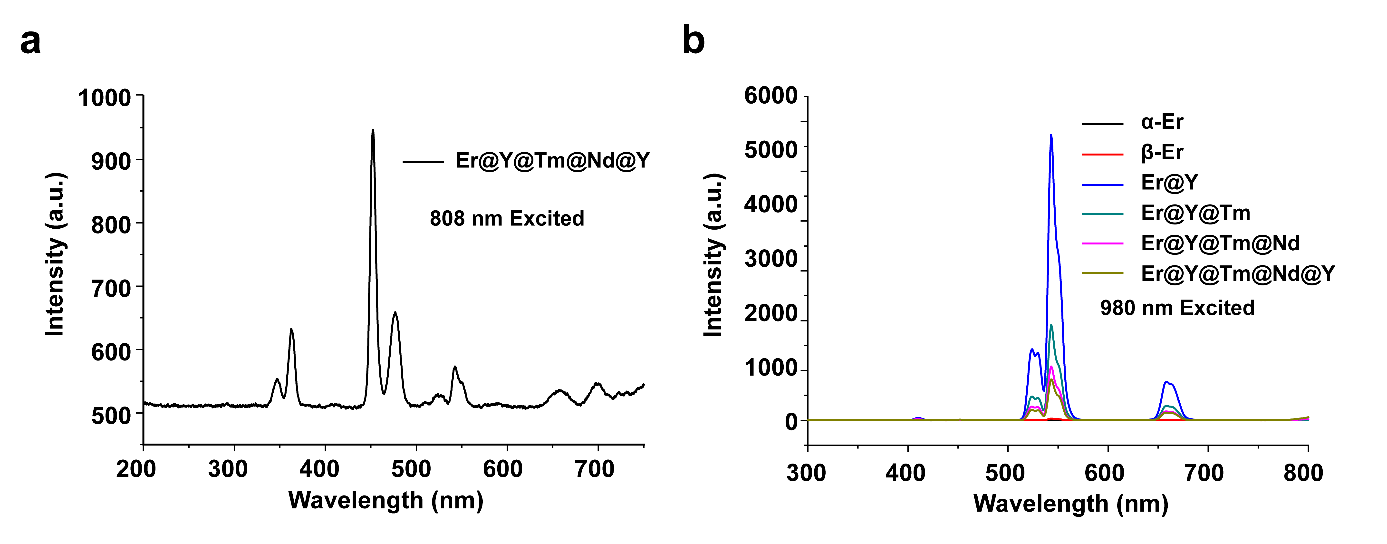


**Figure S6.** (a) UCL spectrum of the core-multishell UCNPs upon excitation of 808 nm. (b) UCL spectrum of the core-multishell UCNPs upon excitation of 980 nm.


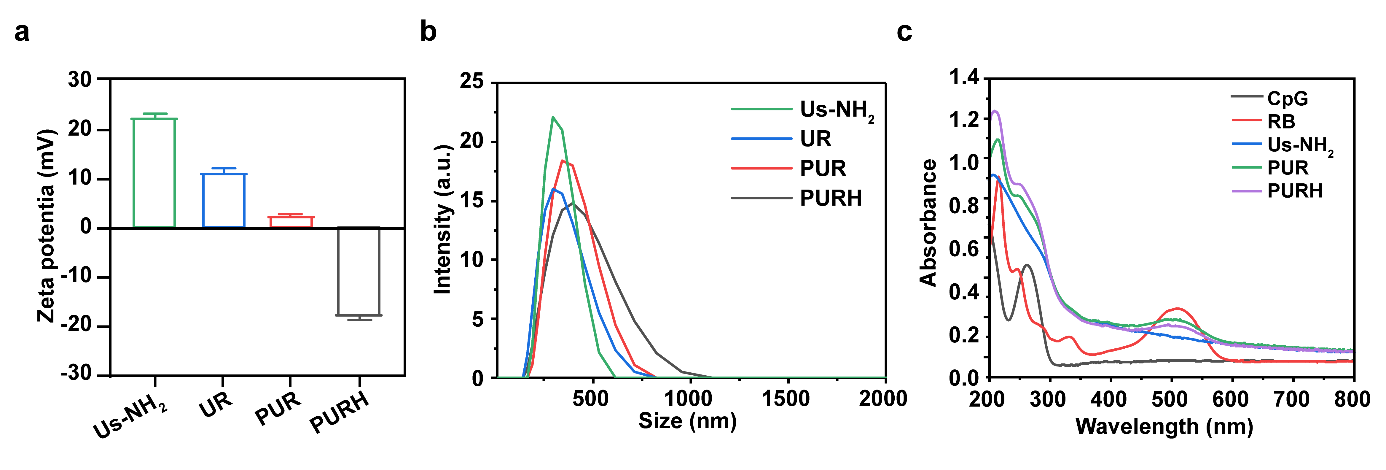


**Figure S7.** (a) Zeta potential of Us-NH_2_ (UCNPs@mSiO_2_-NH_2_), UR, PUR, PURH. Data are presented as mean ± SD (n = 3). (b) Hydrodynamic diameters of Us-NH_2_ (UCNPs@mSiO_2_-NH_2_), UR, PUR, PURH. (c) UV/Vis absorption spectra of Us-NH_2_ (UCNPs@mSiO_2_-NH_2_), PUR, PURH and characteristic peaks of CpG and RB.


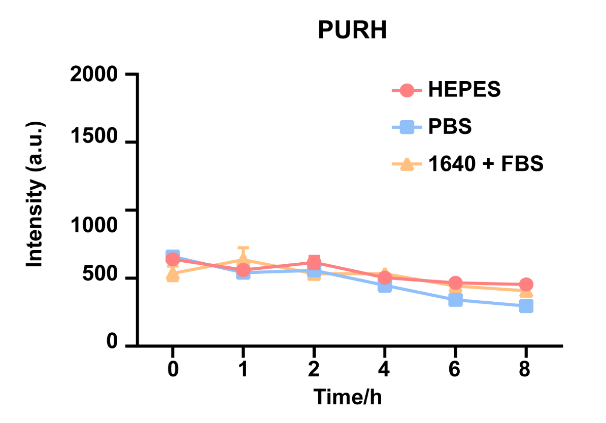


**Figure S8.** Fluorescence intensity of PURH nanoparticle supernatant in HEPES, PBS and RPMI 1640 medium + FBS for different time points. Data are presented as mean ± SD (n = 3).


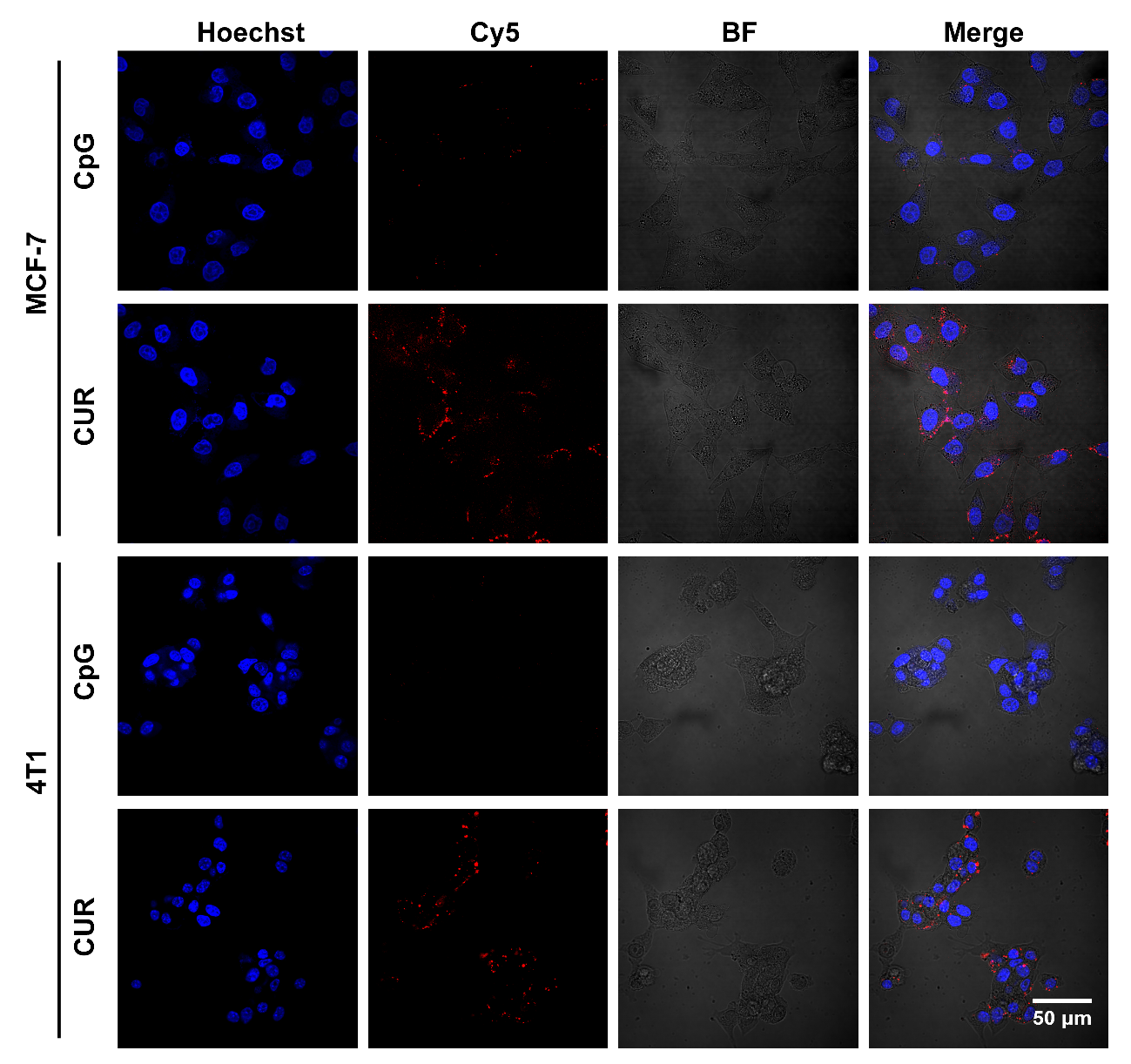


**Figure S9.** CLSM images of 4T1 and MCF-7 cells treated with CpG and CUR (CpG labeled with Cy5). Scale bar, 50 μm.


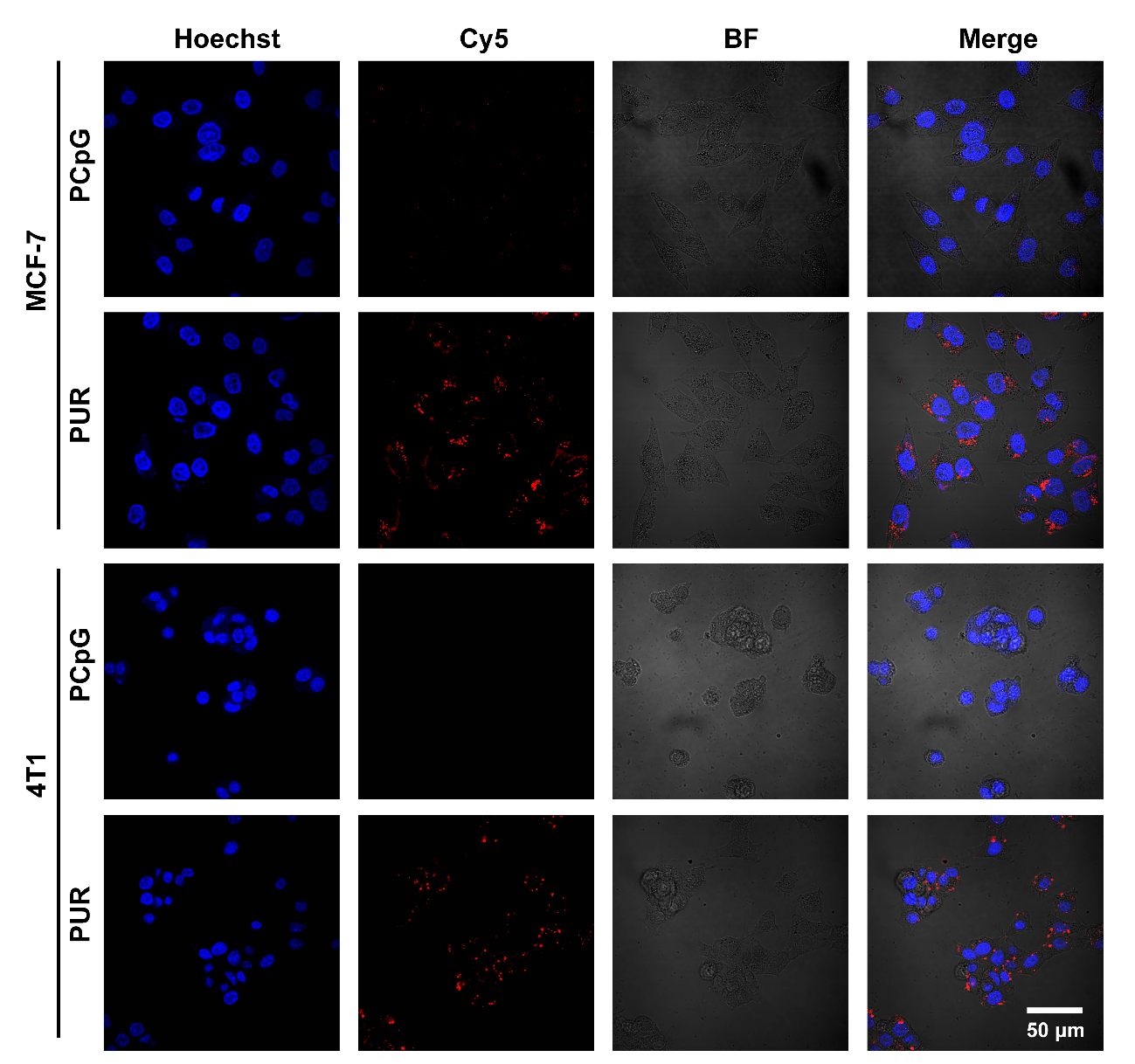


**Figure S10.** CLSM images of 4T1 and MCF-7 cells treated with PCpG and PUR (CpG labeled with Cy5). Scale bar, 50 μm.


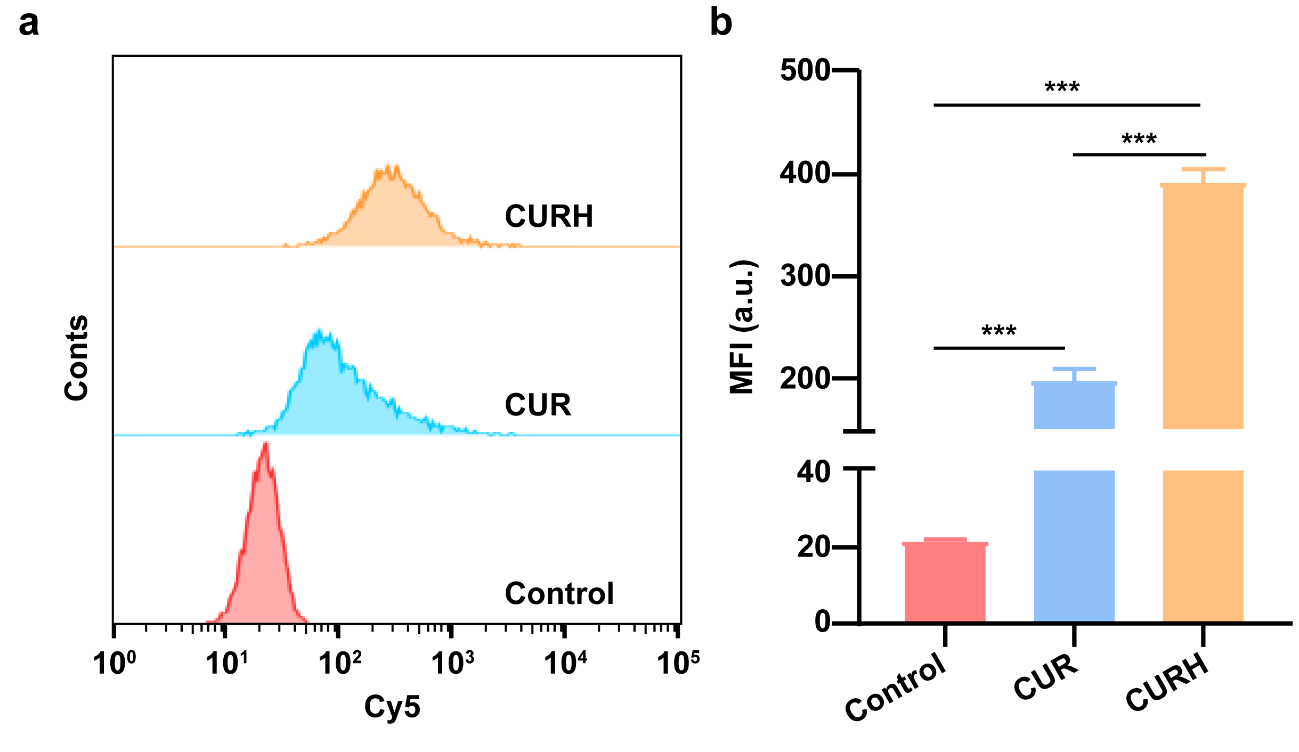


**Figure S11.** (a) Flow cytometry showing the fluorescence of 4T1 cells with CUR and CURH (CpG labeled with Cy5). (b)The quantification of the flow cytometric data. Data are presented as mean ± SD (n = 3). ****P* < 0.001.


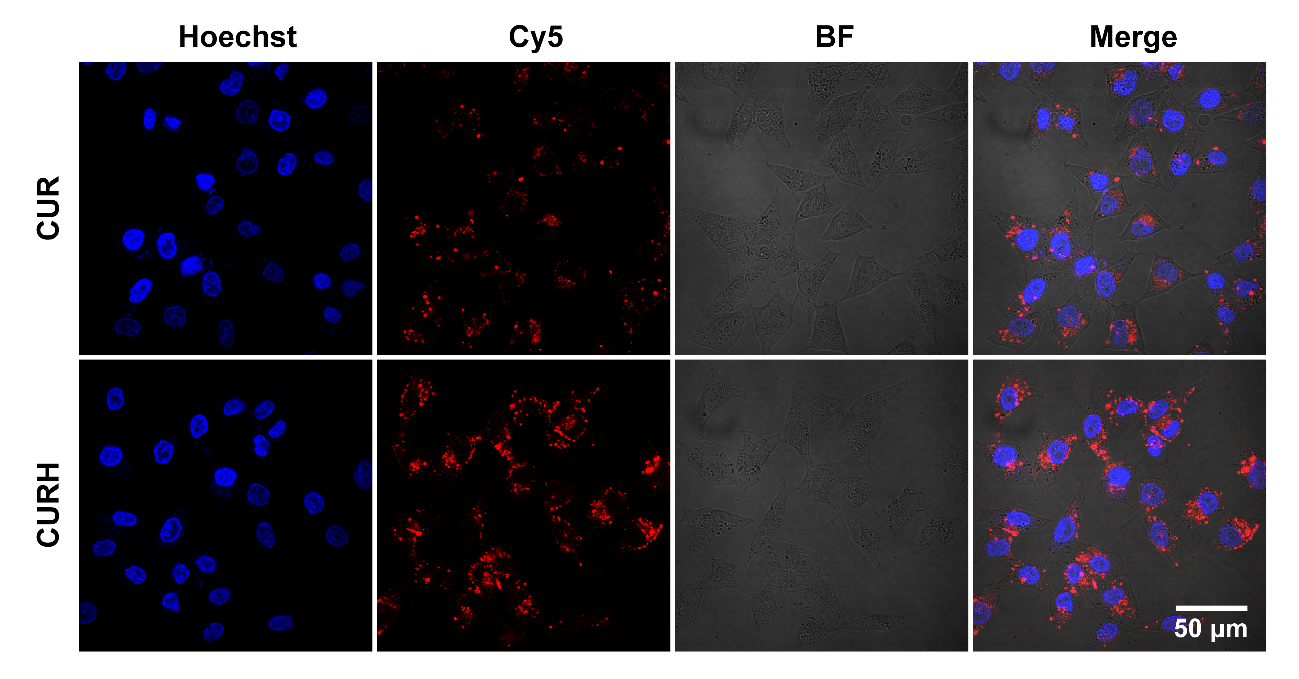


**Figure S12.** CLSM images of MCF-7 cells treated with CUR and CURH (CpG labeled with Cy5). Scale bar, 50 μm.


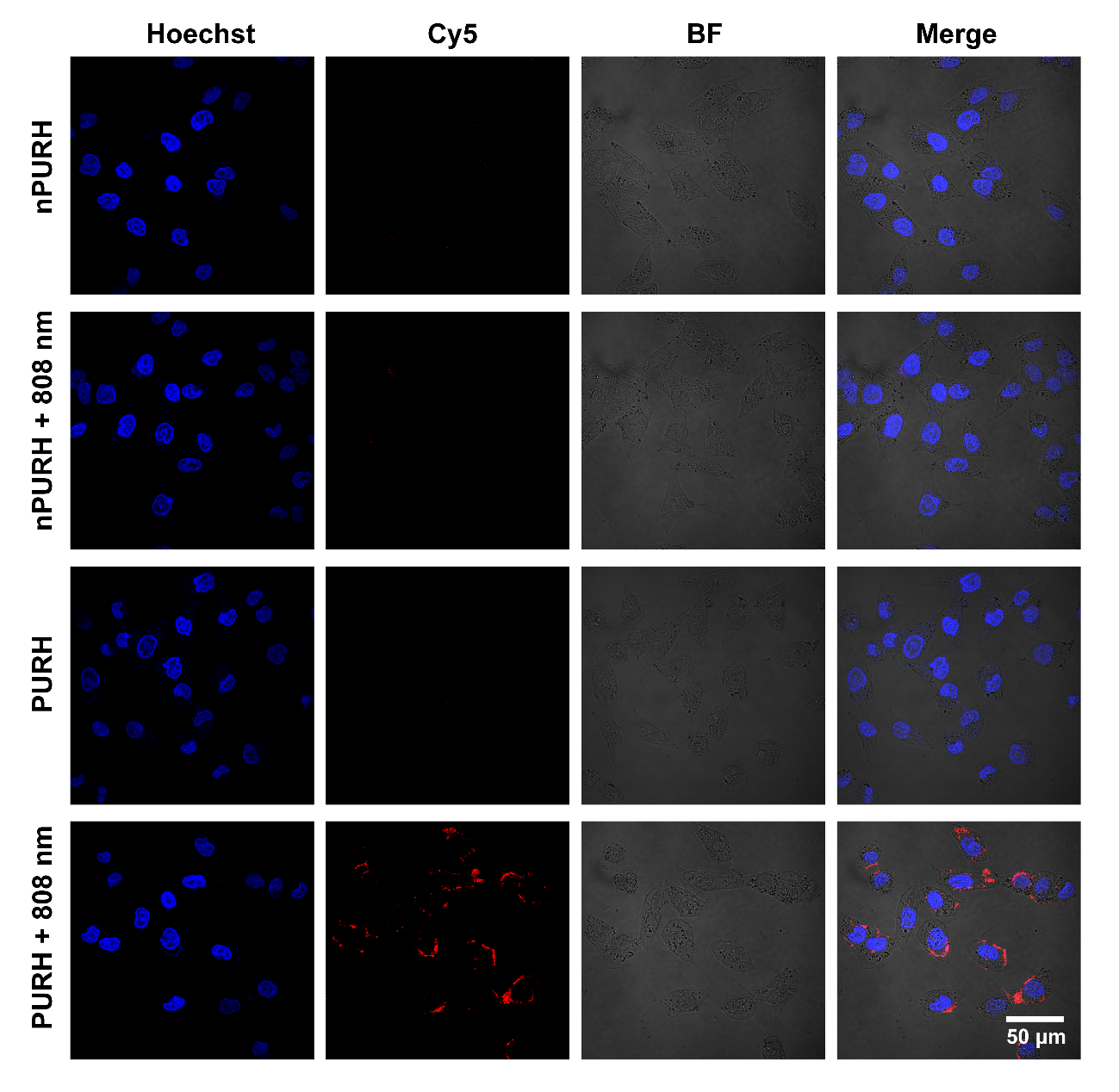


**Figure S13.** CLSM images of MCF-7 cells treated with FRET pair-labled nPURH and PURHT with or without 808 nm NIR light irradiation. Scale, 50 μm.


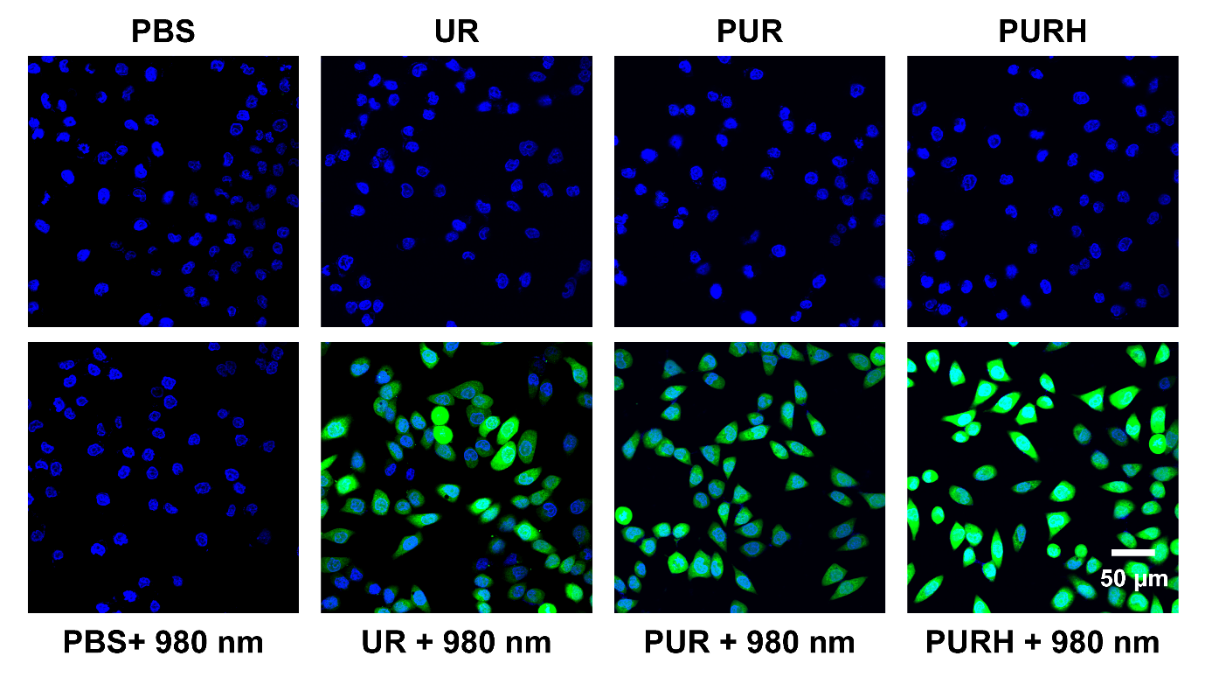


**Figure S14.** CLSM images of DCFH-DA (green) and Hoechst 33342 (blue) showed ROS in MCF-7 cells under different treatments. Scale, 50 μm.


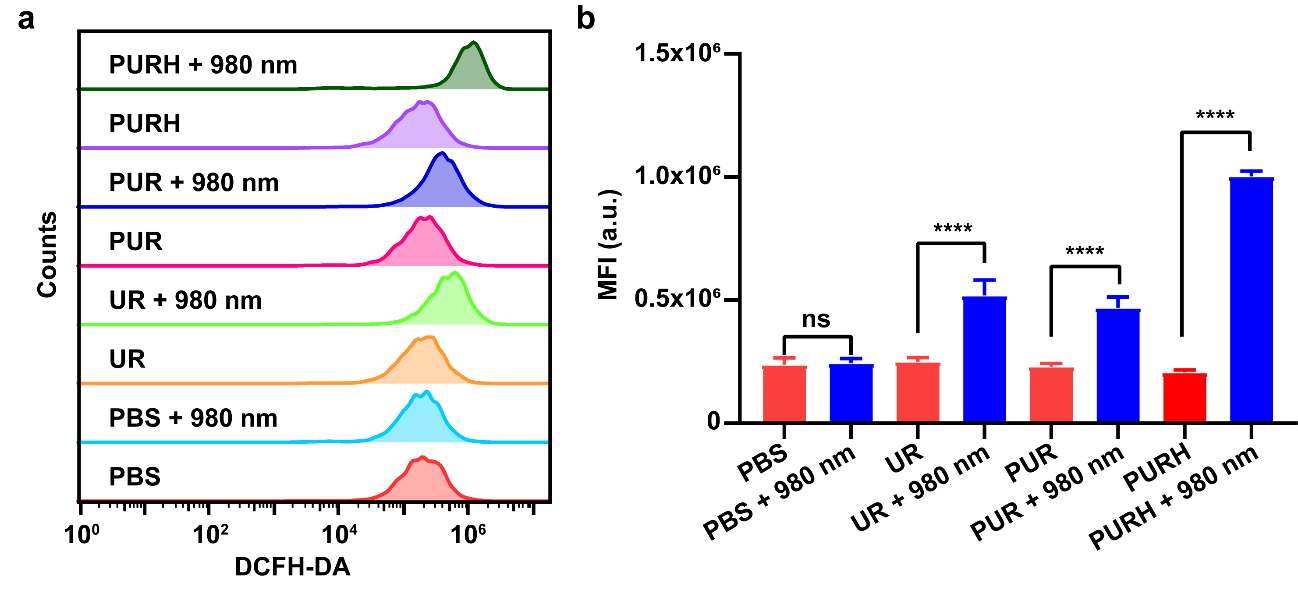


**Figure S15.** (a) Flow cytometry analysis of intracellular ROS of 4T1 cells with different treatments. (b) The quantity analysis of ROS production in 4T1 cells by flow cytometry analysis. Data are presented as mean ± SD (n = 3). *****P* < 0.0001, ns, no significance.


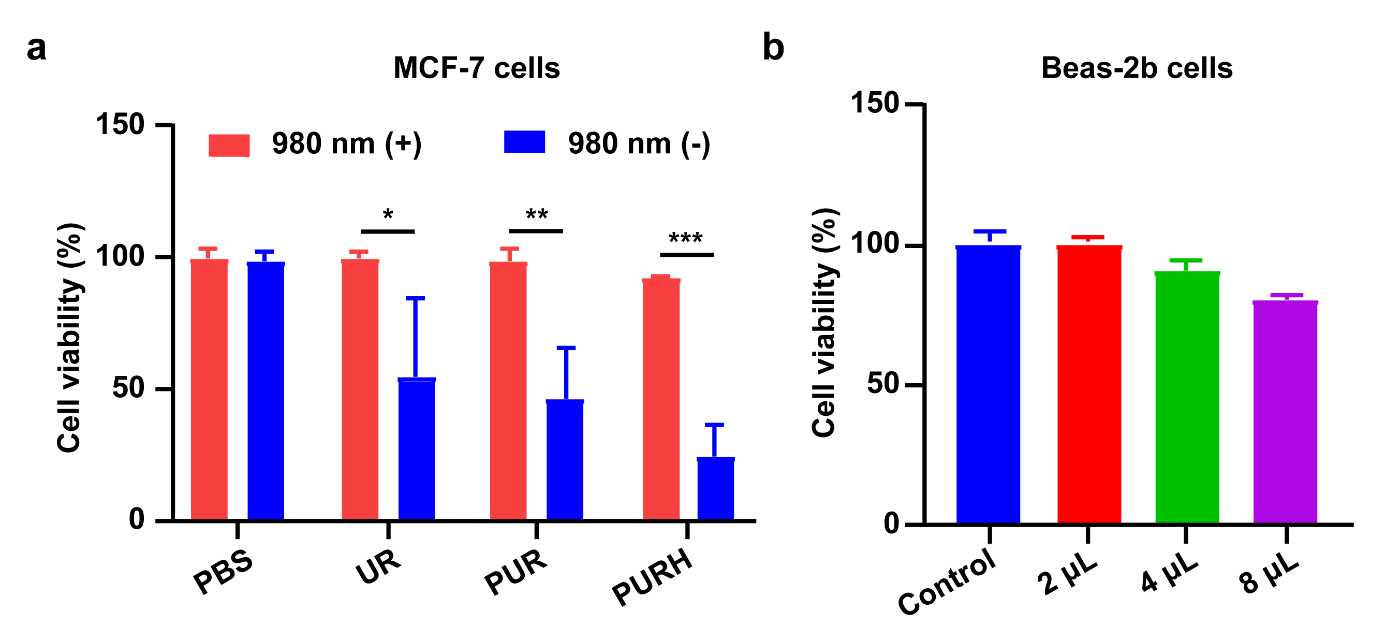


**Figure S16.** (a) Cell viability of MCF-7 cells incubated with different treatments in the dark or with 980 nm NIR light irradiation. Data are presented as the mean ± SD (n = 3). (b) Cell viability of Beas-2b cells incubated with PURH in the dark. Data are presented as the mean ± SD (n = 3). **P* < 0.05, ***P* < 0.01, ****P* < 0.001.


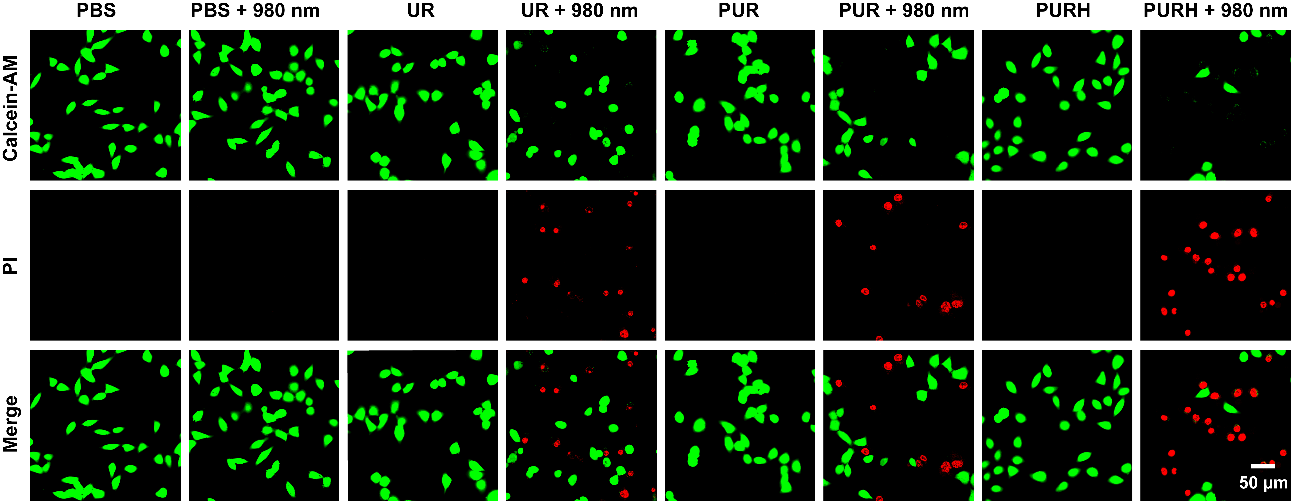


**Figure S17.** Representative confocal fluorescence images of Calcein-AM (green) and PI (red) co-stained MCF-7 cells exposed to different samples with and without 980 nm NIR light irradiation. Scale bar, 50 μm.

**
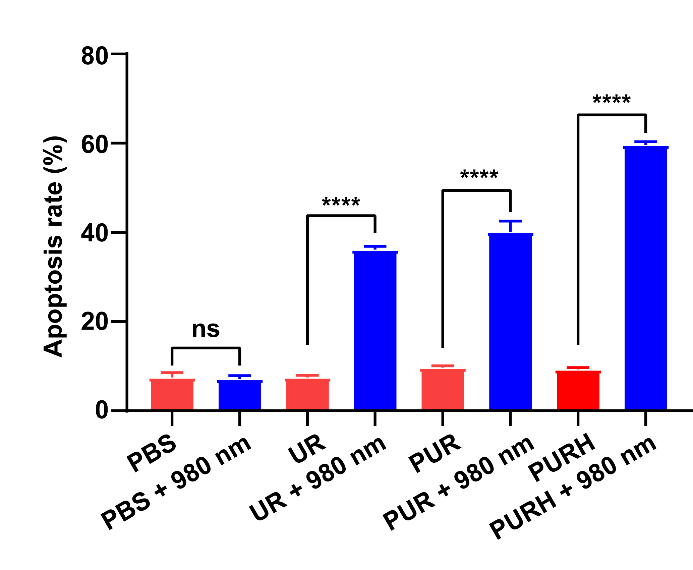
**

**Figure S18.** Cell apoptosis analyzed by flow cytometry with different treatments. Data are presented as mean ± SD (n = 3). *****P* < 0.0001, ns, no significance.


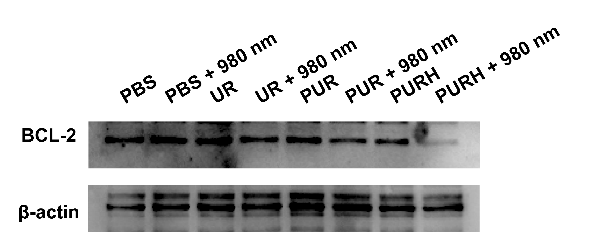


**Figure S19.** Western blotting assay of the expression of BCL-2 protein in 4T1 cells with different treatments.


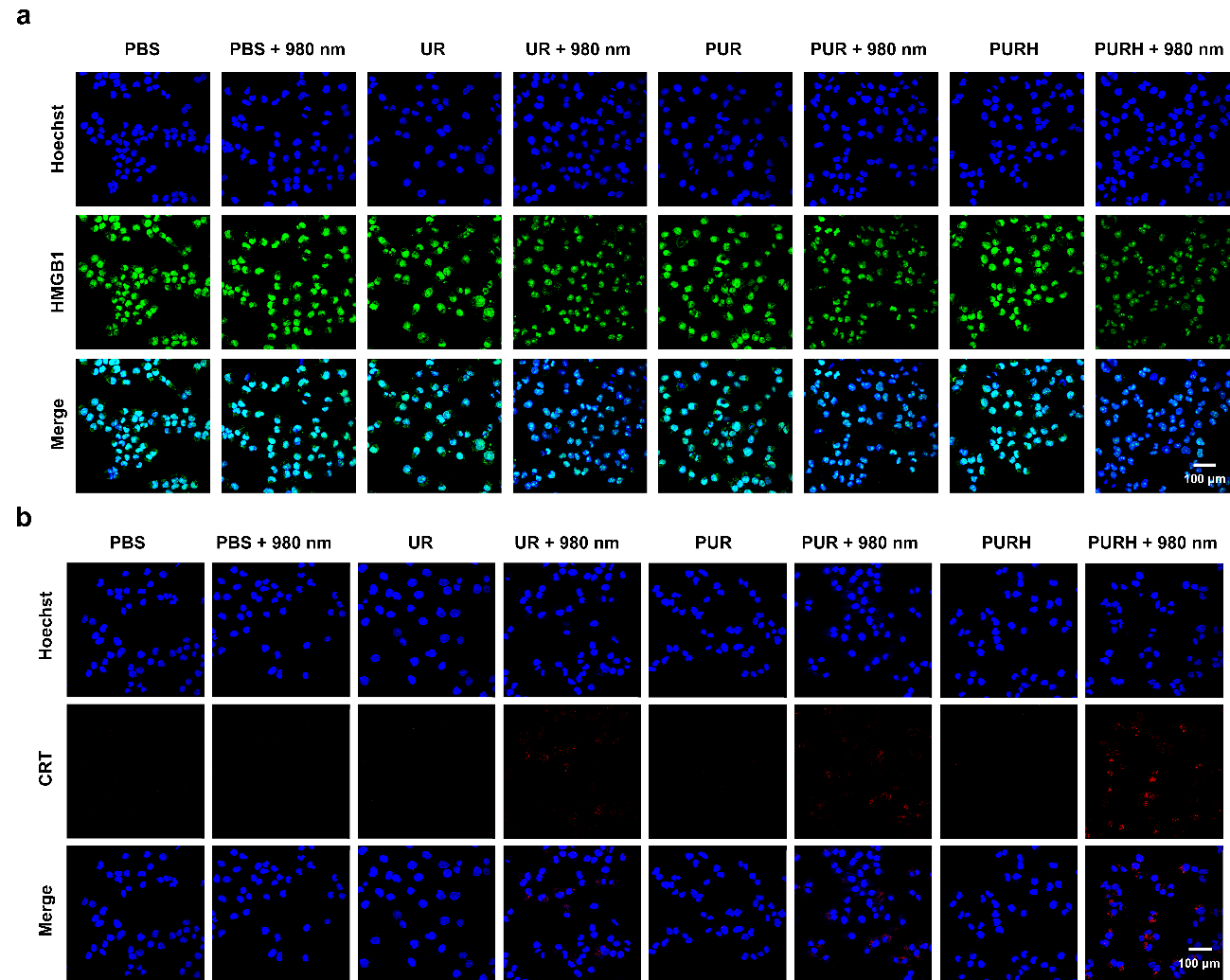


**Figure S20.** Immunofluorescence staining of HMGB1 and CRT in MCF-7 cells treated with different nanomaterials with 980 nm NIR light irradiation. Nuclei were stained blue, HMGB1 was stained green, and CRT was stained red. Scale bar, 50 μm.


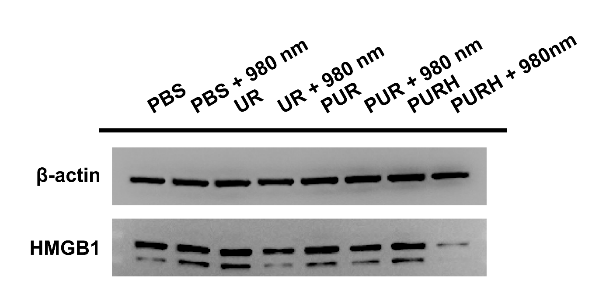


**Figure S21.** Western blot analysis of HMGB1 expression. on 4T1 cells after different treatments with or without 980 nm NIR light irradiation.


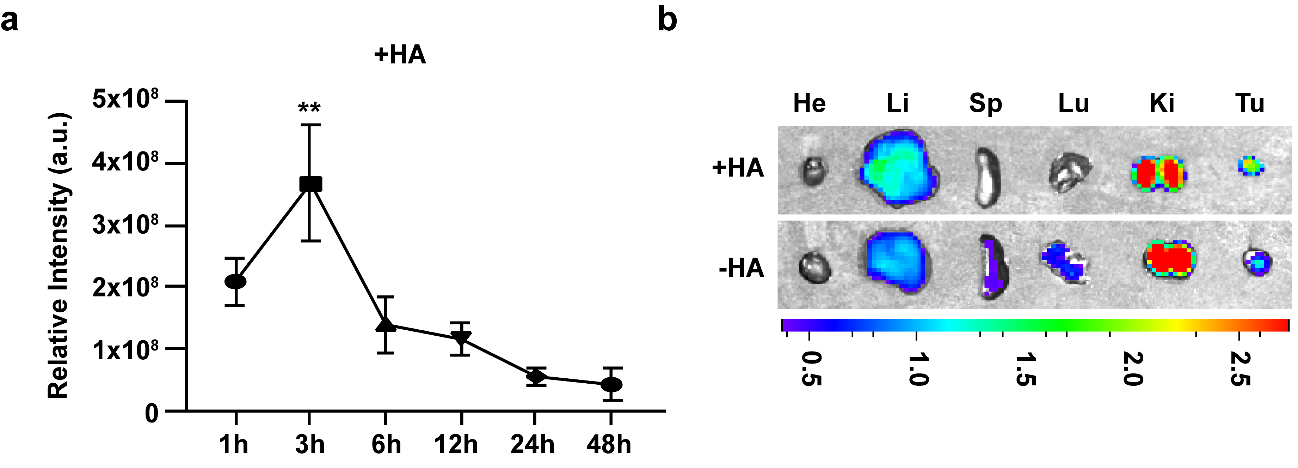


**Figure S22.** (a) Quantitative analysis of fluorescence intensities at tumor sites in mice. Data are presented as the mean ± SD (n = 4). ***P* < 0.01. (b) Ex vivo fluorescence images of major organs and tumors from mice after different treatments. From left to right: heart, liver, spleen, lung, kidney, and tumor.


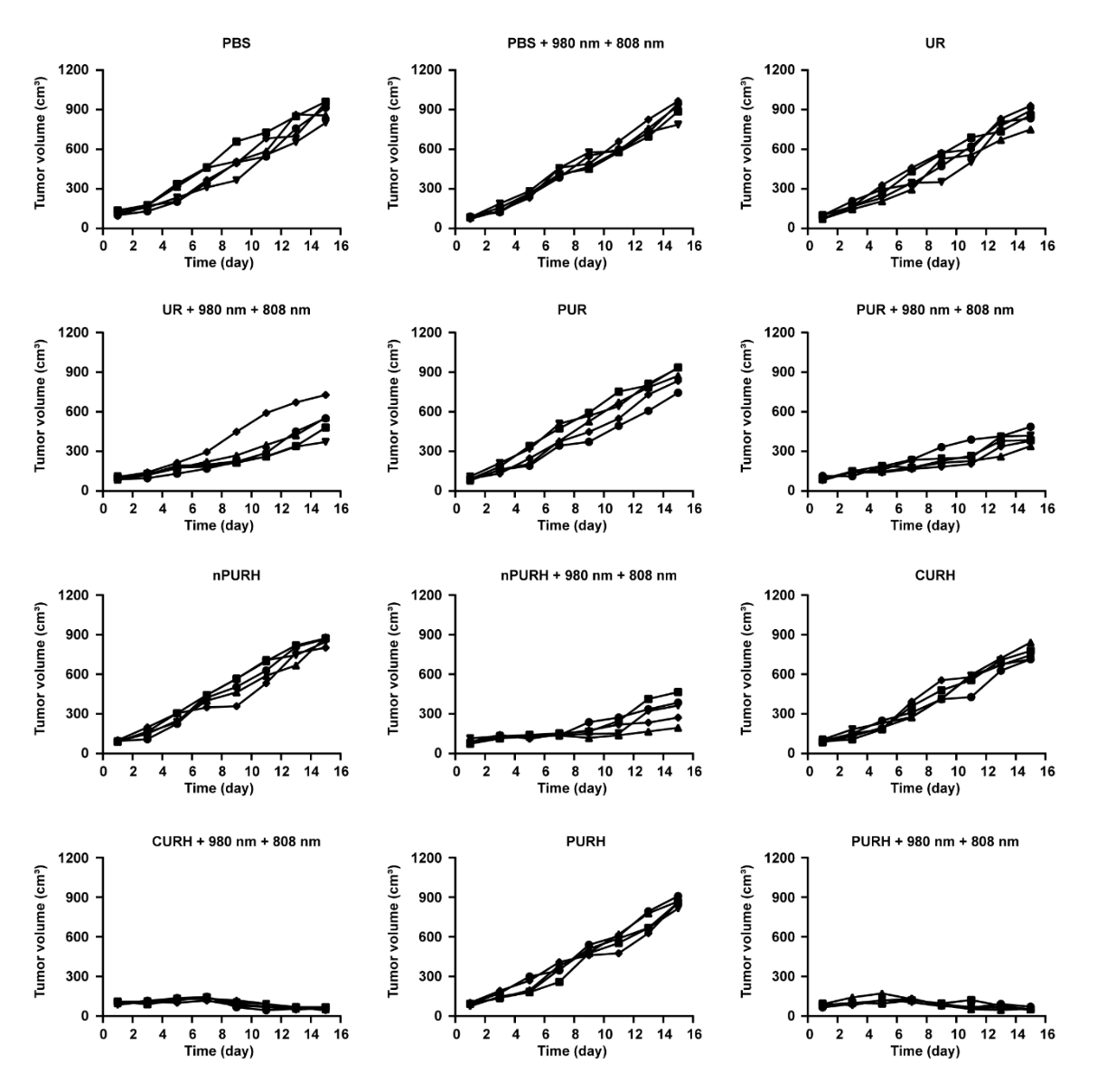


**Figure S23.** Tumor growth curves in each individual 4T1 tumor-bearing mouse with different treatments (n = 5). The data are corresponding to Fig. 5b.


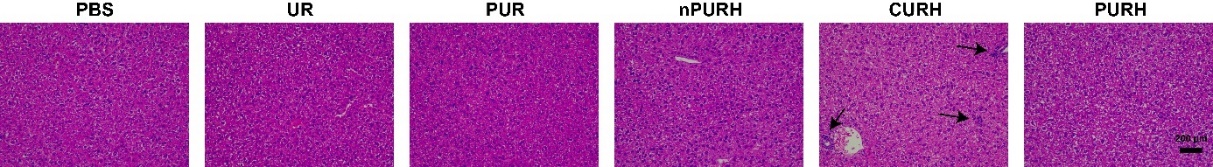


**Figure S24.** Representative H&E-stained tissue sections of live from different treatments mice. Arrows images indicate the inflammation. Scale bar, 200 µm.


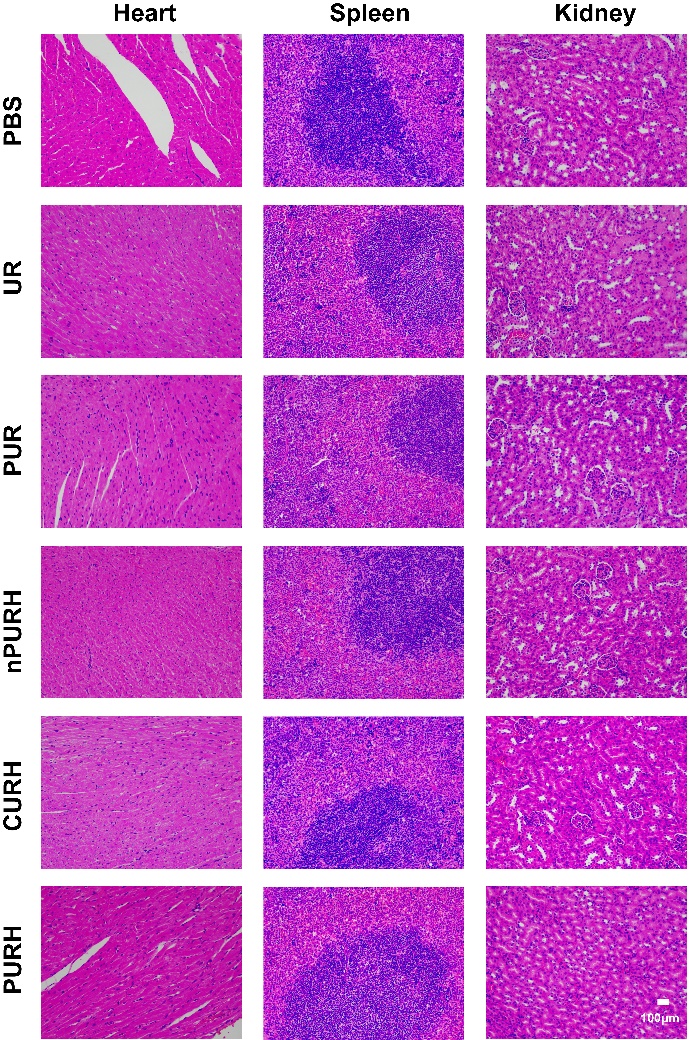


**Figure S25.** Representative H&E-stained tissue sections of heart, lung, spleen and kidney from different treatments mice. Scale bar, 100 µm.


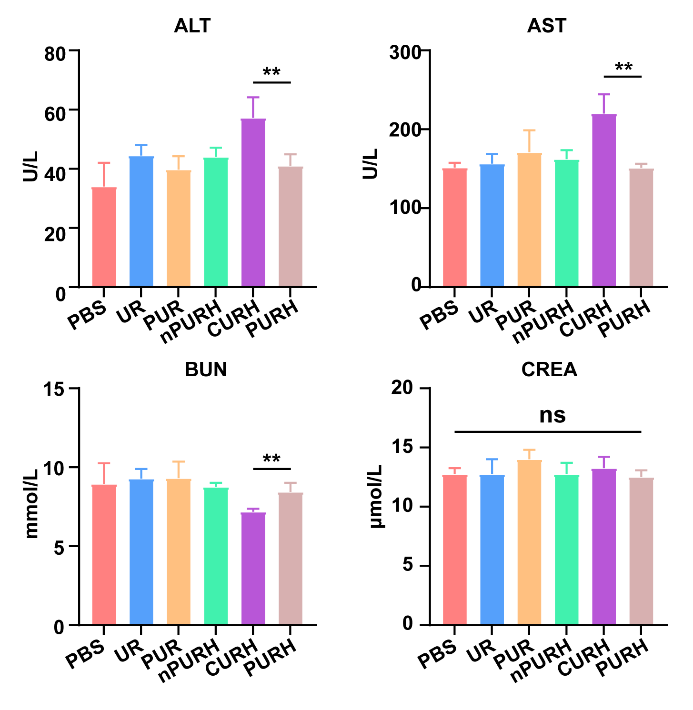


**Figure S26.** Blood biochemical assay of alanine transaminase (ALT), aspartate transaminase (AST), blood urea nitrogen (BUN) and creatinine (CREA) from the mice after different treatments, respectively. Data are presented as mean ± SD (n = 4). ***P* < 0.01, ns = no significance.


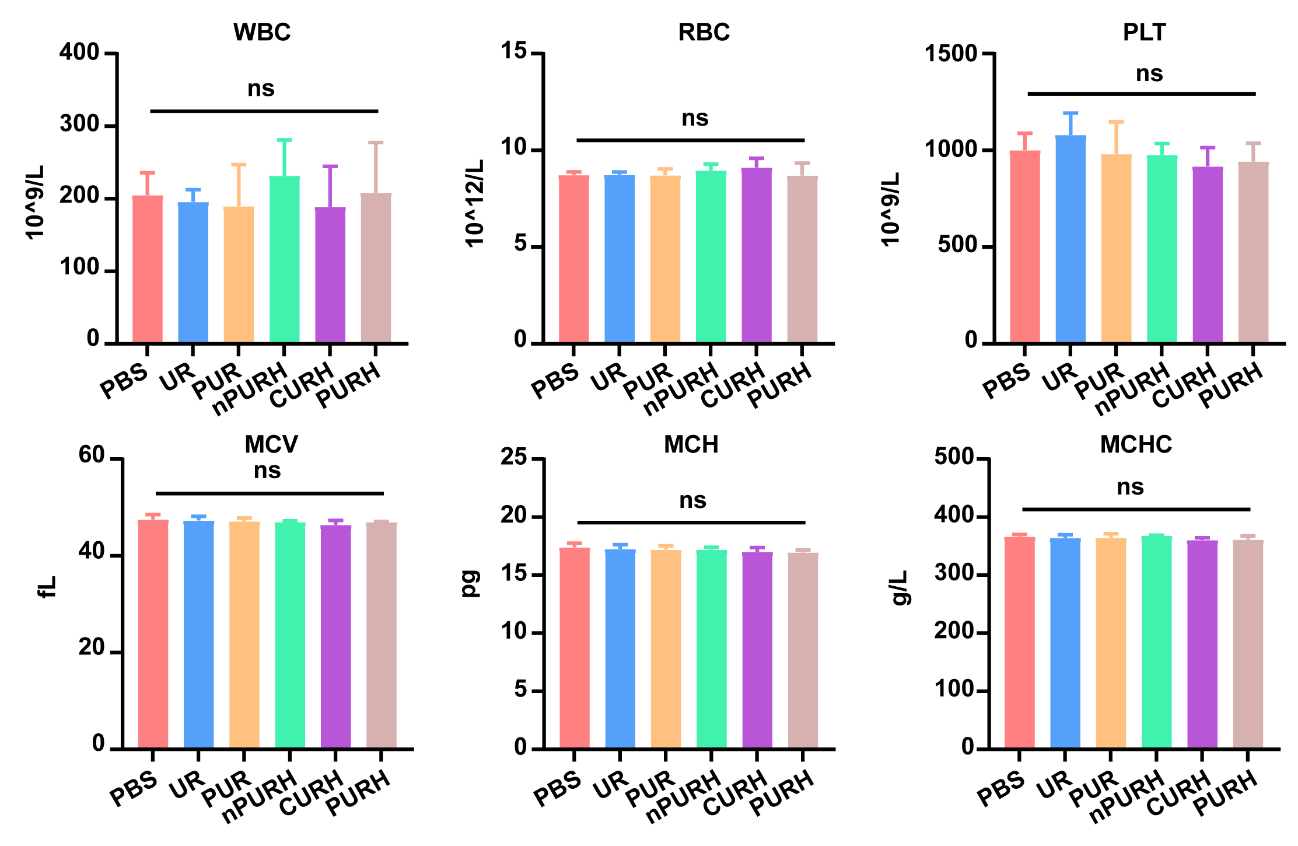


**Figure S27.** Blood routine assessment (white blood cell count: WBC, red blood cell count: RBC, platelet: PLT, mean corpuscular volume: MCV, mean corpuscular hemoglobin: MCH, mean corpuscular hemoglobin concentration: MCHC) to estimate the effects of different groups to blood cells. Data are presented as mean ± SD (n = 4). ns = no significance.


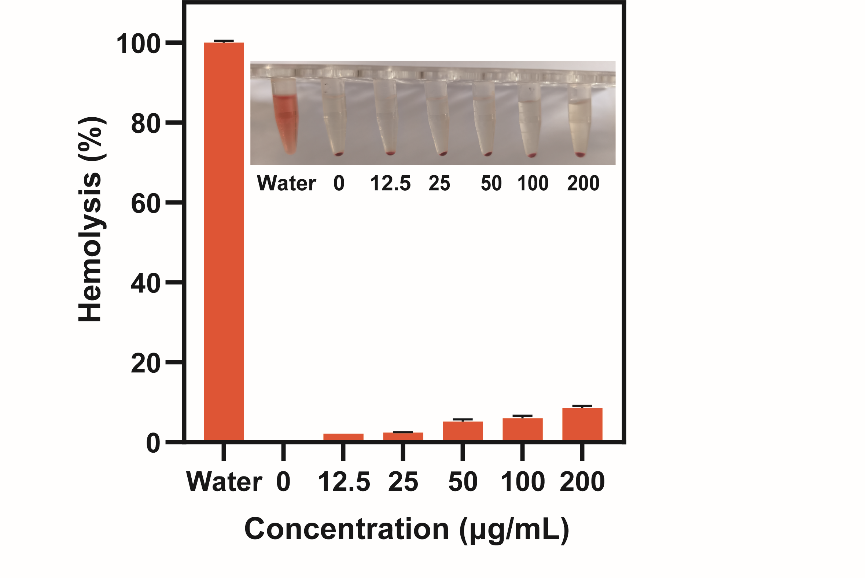


**Figure S28.** Mouse blood samples were treated with various concentrations of PURH to determine hemolysis. PBS (0 μg/mL) served as a negative control, and ddH_2_O served as a positive control. Data are presented as mean ± SD (n = 3)


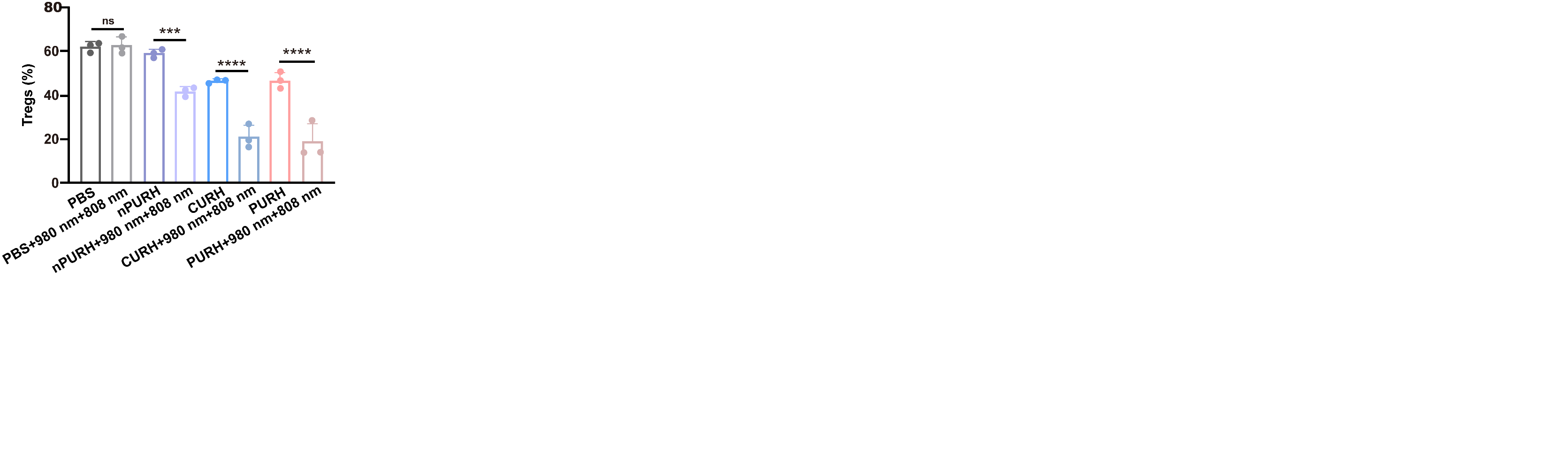


**Figure S29.** Percentage of Tregs (CD3^+^CD4^+^(CD25^+^Foxp3^+^)) in tumors following different treatments. Data are expressed as mean ± SD (n = 3). ****P* < 0.001, *****P* < 0.0001, ns = no significance.


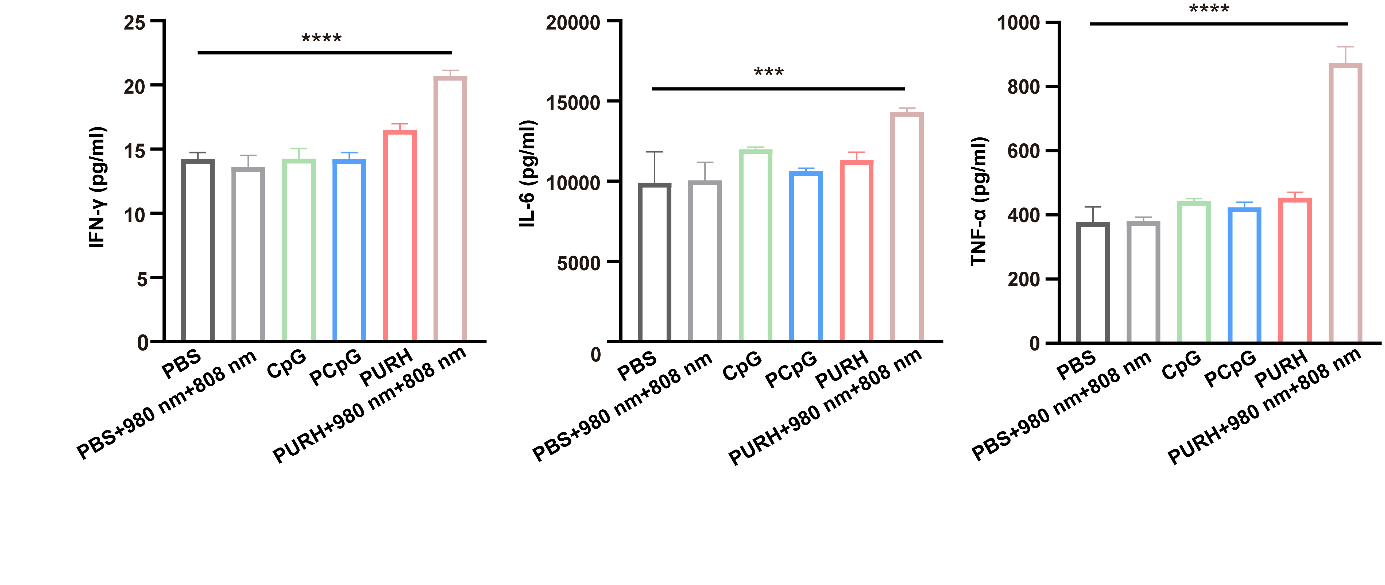


**Figure S30.** Secretion levels of IFN-γ, IL-6 and TNF-α in matured DCs suspensions. Data are presented as means ± SD (n = 3). ****P* < 0.001, *****P* < 0.0001.

**Table S1.** DNA sequence

| Name | Sequence（5’-3’） |
| --- | --- |
| CpG | TCCATGACGTTCCTGACGTT |
| cCpG | AACGTCAGGAACGTCATGGA |
| cCpG-PC | AACGTCAGG/iPC/AACG/iPC/TCATGGA |
| Cy5-CpG | Cy5-TCCATGACGTTCCTGACGTT |
| BHQ-cCpG | AACGTCAGGAACGTCATGGA-BHQ3 |
| BHQ-cCpG-PC | AACGTCAGG/iPC/AACG/iPC/TCATGGA-BHQ3 |

**Table S2.** Full name-abbreviation contrast table

| Full name | Abbreviation |
| --- | --- |
| 1, 3-diphenylisobenofuran | DPBF |
| 2, 7-dichlorodihydrofluorescein diacetate | H_2_DCFDA |
| Adenosine triphosphate | ATP |
| Alanine aminotransferase | ALT |
| Aspartate aminotransferase | AST |
| Calreticulin | CRT |
| Cell counting kit­8 | CCK-8 |
| Confocal laser scanning microscope | CLSM |
| CpG/UCNP@mSiO_2_-RB-HA | CURH |
| CpG/UCNPs@mSiO_2_-RB | CUR |
| Cyanine5 | Cy5 |
| Damage associated molecular patterns | DAMPs |
| Dendritic cells | DCs |
| Energy dispersive spectroscopy | EDS |
| Enzyme-linked immunosorbent assay | ELISA |
| Förster resonance energy transfer | FRET |
| Fourier transform infrared | FTIR |
| Hematoxylin and eosin | H&E |
| High mobility group protein | HMGB1 |
| High­angle annular dark­field scanning TEM | HAADF-STEM |
| Hyaluronic acid | HA |
| Immunogenic cell death | ICD |
| *In vivo* imaging system | IVIS |
| Near-infrared | NIR |
| nPCpG/UCNP@mSiO_2_-RB-HA | nPURH |
| PCpG/UCNP@mSiO_2_-RB-HA | PURH |
| PCpG/UCNPs@mSiO_2_-RB | PUR |
| Photo-controlled | PC |
| Photodynamic therapy | PDT |
| Photosensitizers | PSs |
| Reactive oxygen species | ROS |
| Rose bengal | RB |
| Single oxygen | ^1^O_2_ |
| Toll-like receptor 9 | TLR-9 |
| Tumor microenvironment | TME |
| Tumor-associated antigens | TAAs |
| UCNPs@mSiO_2_-RB | UR |
| Ultraviolet | UV |
| Upconversion luminescence | UCL |
| Upconversion nanoparticles | UCNPs |
| Visible | Vis |
